# Maximum likelihood estimation based on Newton–Raphson iteration for the bivariate random effects model in test accuracy meta-analysis

**DOI:** 10.1177/0962280219853602

**Published:** 2019-06-11

**Authors:** Brian H Willis, Mohammed Baragilly, Dyuti Coomar

**Affiliations:** 1 Institute of Applied Health Research, University of Birmingham, Birmingham, UK; 2 Department of Applied Statistics, Helwan University, Cairo, Egypt

**Keywords:** Bivariate model, diagnostic accuracy, maximum likelihood estimation, meta-analysis, random effects

## Abstract

A bivariate generalised linear mixed model is often used for meta-analysis of test accuracy studies. The model is complex and requires five parameters to be estimated. As there is no closed form for the likelihood function for the model, maximum likelihood estimates for the parameters have to be obtained numerically. Although generic functions have emerged which may estimate the parameters in these models, they remain opaque to many. From first principles we demonstrate how the maximum likelihood estimates for the parameters may be obtained using two methods based on Newton–Raphson iteration. The first uses the profile likelihood and the second uses the Observed Fisher Information. As convergence may depend on the proximity of the initial estimates to the global maximum, each algorithm includes a method for obtaining robust initial estimates. A simulation study was used to evaluate the algorithms and compare their performance with the generic generalised linear mixed model function *glmer* from the *lme4* package in R before applying them to two meta-analyses from the literature. In general, the two algorithms had higher convergence rates and coverage probabilities than *glmer*. Based on its performance characteristics the method of profiling is recommended for fitting the bivariate generalised linear mixed model for meta-analysis.

## 1 Introduction

Meta-analysis may be used to aggregate data from multiple primary studies to produce summary estimates. The most common type of model used in meta-analysis involves aggregating data where a single outcome measure is used to summarise the effect measure. Such univariate modelling approaches have yielded notable successes for meta-analysis where the results have helped inform medical decisions on treatments of life threatening diseases.^1,2^

In the case of meta-analysis of test accuracy studies, the picture is complicated by there being, in general, two outcomes of interest that are correlated. The modelling approach taken in this instance is to assume the study-level parameters for the outcomes follow a bivariate normal distribution.^3,4^ Although, after a suitable transformation, we may assume the observed data within studies to be normally distributed,^3^ this is an approximation and they are more accurately modelled by assuming binomial distributions.^4^ Thus, to aggregate the data from test accuracy studies, a bivariate generalised linear mixed model is used. Note it is more commonly labelled a bivariate random effects model (BRM)^3^ and this will be the term which will be adopted here when referring to the model.

As with many complex models of this nature, there is no closed form to the likelihood function for the model, so it is not possible to express the maximum likelihood estimates (MLEs) for the parameters analytically and numerical solutions are required. Although some packages are capable of providing maximum likelihood estimates for the parameters in the BRM, they tend to be generic packages in which the algorithms are not readily accessible and are not necessarily optimised for this model. For example, the *glmer* function from the *lme4* package in R^5^ and *NLMIXED* in SAS^6^ are used to fit a range of generalised linear and non-linear models and are not specifically written for estimating the parameters in the BRM. Thus, an algorithm which is expressly written and optimised to fit the BRM has the potential for better performance characteristics than that of a generic function. It also needs to be transparent in order to facilitate understanding and reproducibility.

Here we develop two different optimisation approaches based on Newton–Raphson methods,^7^ specifically to derive the maximum likelihood estimates for the parameters in the BRM. To demonstrate how this may be done from first principles, the theory and steps behind the optimisation are described explicitly, and the R code is provided in the online Appendix. We conduct a simulation study to evaluate the two algorithms and compare their performances with that of a generic function from a standard package, namely, the *glmer* function in the *lme4* package in R.^5^ We then apply the algorithms to two case examples.

The paper is organised as follows. In section 2, we describe the theory in detail that underpins the bivariate random effects model used in test accuracy meta-analyses. In section 3, the optimisation methods in generic packages that may be used to fit the BRM are described. In section 4, the theory behind deriving maximum likelihood estimates in the BRM is explained in detail. In sections 5 and 6, the method of Profiling^8^ and the Observed Fisher Information using robust initial parameter values (OFIRIV) are developed for the BRM. In section 7, these methods are compared using a simulation study and applying them to two case examples from the literature. Finally, in section 8, we end with the discussion.

## 2 Statistical methodology

A test's performance is traditionally summarised in terms of its sensitivity (the proportion of patients with disease who test positive) and specificity (the proportion of patients without disease who test negative). The two are also correlated being affected by the position of the threshold for a positive test result: as the threshold increases, the sensitivity decreases and the specificity increases. This effect is summarised by a receiver operating characteristic (ROC) curve which plots the different sensitivity–specificity pairs for each test threshold.^9^

An early attempt to incorporate such an effect in meta-analysis was made by Moses and colleagues,^10^ who produced a Summary ROC (SROC) curve using simple linear regression. The model does capture variation between studies due to a changing threshold but other sources of variation are largely ignored. For the purpose of translation into practice, a summary point is usually more desirable but a valid point estimate is not readily provided by this model.

Attempts to overcome these limitations^3,4^ have led to the proposing of hierarchical models.^3,4,11^ Van Houwelingen^12^ applied a bivariate random effects model to meta-analysis which was later taken up by Reitsma,^3^ who applied it to test accuracy meta-analyses. This model allows a summary point for the sensitivity and specificity in ROC space to be estimated. An alternative approach as proposed by Rutter and Gatsonis^9^ leads to a Hierarchical Summary Receiver Operating Characteristic (HSROC) curve, although a summary point may be derived from this model. Here we will focus on the bivariate random effects model for test accuracy studies. The model is a mixed model and assumes a bivariate normal distribution of the form

(1) $$\begin{array}{c} (\begin{array}{c} \alpha_{i}\\ \beta_{i} \end{array})∼N((\begin{array}{c} \alpha \\ \beta \end{array}),(\begin{array}{cc} \sigma_{A}^{2} & \sigma_{\mathrm{AB}}\\ \sigma_{\mathrm{AB}} & \sigma_{B}^{2} \end{array})) \end{array}$$

where $\alpha_{i}$ and $\beta_{i}$ are the logit sensitivity and logit specificity for the *i*^th^ study, α and $\sigma_{A}^{2}$ are the mean and variance for the logit sensitivities, β and $\sigma_{B}^{2}$ are the mean and variance for the logit specificities, and $\sigma_{\mathrm{AB}}$ is the covariance between $\alpha_{i}$ and $\beta_{i}$ across studies, respectively. In some of the literature, it is common to replace the covariance term $\sigma_{\mathrm{AB}}$ by the multiplication $\rho \sigma_{A}\sigma_{B}$ to include the correlation ρ in the model,^4^ so the covariance matrix in equation (1) can be written as

(2) $$\Sigma =(\begin{array}{cc} \sigma_{A}^{2} & \rho \sigma_{A}\sigma_{B}\\ \rho \sigma_{A}\sigma_{B} & \sigma_{B}^{2} \end{array})$$

Thus, the five parameters $\left(\alpha ,\beta ,\sigma_{a}^{2},\sigma_{b}^{2},\rho \right)$ need to be estimated in order to make inferences on the sensitivity and specificity.

For a test accuracy review with *k* studies, let $TP_{i}$, $TN_{i}$, $n_{A,i}$ and $n_{B,i}$ be the number of true positives, true negatives, diseased, and non-diseased for the *i*^th^ study, respectively. Chu and Cole^4^ pointed out that a binomial likelihood should be used for modelling within-study variability especially if the data are sparse, so the model should include the following components

(3) $$TP_{i}|P_{A,i}∼\mathrm{Binomial}(n_{A,i},P_{A,i})$$

(4) $$TN_{i}|P_{B,i}∼\mathrm{Binomial}(n_{B,i},P_{B,i})$$

where $P_{A,i}$ and $P_{B,i}$ represent the study-specific sensitivity and specificity, respectively. If both $P_{A,i}$ and $P_{B,i}$ are known, then $TP_{i}$ and $TN_{i}$ are assumed to follow independent binomial distributions.^4,13^ In the random effects models, we assume that each study has its own test sensitivity and specificity, in other words the model includes a between-study variance component and correlation between $P_{A,i}$ and $P_{B,i}$, such that

(5) $$\begin{array}{c} g(P_{A,i})=X_{i}^{T}\alpha +\alpha_{i},g(P_{B,i})=Z_{i}^{T}\beta +\beta_{i} \end{array}$$

where $X_{i}$ is a vector of study-level covariates for $P_{A,i}$ and $Z_{i}$ is a vector of study-level covariates for $P_{B,i}$ and both $\alpha_{i},\beta_{i}$ are supposed to follow a bivariate normal distribution defined in equation (1). Although the logit link function g(.) is commonly used in equation (5), other link functions can be applied. However, we will use the logit link function g(.) and assume that $X_{i}=Z_{i}=1$ in equation (5) throughout, so α and β will be the respective overall logit sensitivity and logit specificity.

The parameters of the bivariate generalised linear mixed effect model may be estimated by maximising the likelihood function. The log-likelihood function, $l(\alpha ,\beta ,\sigma_{A}^{2},\sigma_{B}^{2},\rho )$ for the model may be written as

(6) $$\begin{array}{c} l(\alpha ,\beta ,\sigma_{A}^{2},\sigma_{B}^{2},\rho )=\text{log}\overset{k}{\underset{i=1}{\Pi }}p_{r}(TP_{i},TN_{i}|n_{A,i},n_{B,i})=\sum_{i=1}^{k}\text{log}p_{r}(TP_{i},TN_{i}|n_{A,i},n_{B,i})\\ =\sum_{i=1}^{k}\text{log}\int \int \mathrm{Bin}(TP_{i}|n_{A,i};P_{A,i})\mathrm{Bin}(TN_{i}|n_{B,i};P_{B,i})\varphi (P_{A,i},P_{B,i};\alpha ,\beta ,\sigma_{A}^{2},\sigma_{B}^{2},\rho )\text{d}P_{A,i}\text{d}P_{B,i} \end{array}$$

where

(7) $$\begin{array}{c} \mathrm{Bin}(TP_{i}|n_{A,i};P_{A,i})=(\begin{array}{c} n_{A,i}\\ TP_{i} \end{array}){P_{A,i}}^{TP_{i}}(1-P_{A,i}{)}^{n_{A,i}-TP_{i}} \end{array}$$

(8) $$\mathrm{Bin}(TN_{i}|n_{B,i};P_{B,i})=(\begin{array}{c} n_{B,i}\\ TN_{i} \end{array}){P_{B,i}}^{TN_{i}}(1-P_{B,i}{)}^{n_{B,i}-TN_{i}}$$

and $\varphi =\varphi (P_{A,i},P_{B,i};\alpha ,\beta ,\sigma_{A}^{2},\sigma_{B}^{2},\rho )$ is the bivariate logit normal distribution, such that

(9) $$\begin{array}{c} \varphi =Ke^{\{-\frac{1}{2(1-\rho^{2})}[\frac{(\mathrm{logit}(P_{A,i})-\alpha )2}{{\sigma_{A}}^{2}}+\frac{(\mathrm{logit}(P_{B,i})-\beta )2}{{\sigma_{B}}^{2}}-\frac{2\rho (\mathrm{logit}(P_{A,i})-\alpha )(\mathrm{logit}(P_{B,i})-\beta )}{\sigma_{A}\sigma_{B}}]\}} \end{array}$$

where

$$\begin{array}{c} K=\frac{1}{2\pi \sigma_{A}\sigma_{B}\sqrt{1-\rho^{2}}P_{A,i}(1-P_{A,i})P_{B,i}(1-P_{B,i})} \end{array}$$

From inspecting the log likelihood function in equation (6), it can be seen that it involves a double integration over the random effects and there is no closed form so it cannot be solved analytically. In order to get a solution to the integral, we have to use numerical optimisation methods such as the Laplacian approximation or the adaptive Gaussian quadrature^14^ to evaluate this integral. Before proceeding to derive the maximum likelihood estimates of the BRM using methods based on the Newton-Raphson algorithm,^7^ we will briefly describe the optimisation approaches used in two generic packages.

## 3 Optimisation methods used in generic packages

Both the *glmer* function in the *lme4* package in R^5^ and the *NLMIXED* function in SAS^6^ are generic functions that have been developed to optimise a range of generalised mixed and non-linear mixed models. As such they may be used to provide estimates for the bivariate generalised linear mixed model or BRM. Both use a Cholesky parameterisation of the models being optimised.^5,6^

Briefly, one of the issues in estimating the parameters in any generalised mixed model is that the covariance matrix of random effects, Σ(θ), may be singular and thus its inverse may not exist. In some cases, this may be overcome by re-formulating the objective function. Thus, for random effects vector V, Σ(θ) may be re-formulated in terms of a relative covariance factor Λ(θ), for a variance component θ, allowing V to be expressed as the product Λ(θ)U, where U is a spherical random effects vector. Taking this approach, the likelihood function may be written in terms of sparse Cholesky factors and finding the maximum likelihood is transformed into finding the penalised least squares.^5,15^ By writing the likelihood in terms of sparse Cholesky factors, the problem may be reformulated so that the resulting matrix is not singular even when Σ(θ) is singular.^15^

This is the approach taken in the *glmer* function in the *lme4* package in R^5^ and the initial values of θ for the sparse Cholesky factors are taken to be 1 on the diagonal and 0 for off diagonal elements.^16^

The default numerical optimisation algorithms used in *glmer* are the Nelder–Mead and the Bounded Optimisation By Quadratic Approximation (BOBYQA).^17^ The Nelder–Mead method is a derivative-free optimisation (DFO) algorithm^18^ introduced as a means of optimising functions when the derivatives are not available or unknown. It starts with a simplex (a generalisation of a triangle to *n* dimensions) so that a function of *n* variables is evaluated at *n* + 1 points. The values of the function at these points are ranked and by geometric transformations (reflection, contraction, and expansion) the point where the function is largest is replaced with a point where the function is smaller. This gives a new simplex and the process continues until convergence.

The BOBYQA algorithm is a sophisticated algorithm and one of several due to Powell which is derivative free.^19^ Essentially it is based on using a quadratic model to locally approximate the objective function, F, over a trust region. After *k* iterations, the coefficients of the quadratic model *Q*_*k*_ are obtained by constraining *Q*_*k*_ to interpolate F at a fixed number of points – these are the interpolation conditions. The sub-problem is to find *d*_*k*_ such that *x_k_ + d_k_* minimises *Q*_*k*_ over the trust region. If *x_k_ + d_k_* improves on the current iterate *x*_*k*_, then this becomes the new iterate *x*_*k+1*_ and the trust region and quadratic model *Q*_*k*_ are updated. If it is not an improvement, then an alternative iteration algorithm is used to identify *d*_*k*_ so that it ensures linear independence in the interpolation conditions. Broadly, this process continues until convergence.

Other derivative approaches may be used to fit the bivariate model as is the case with *NLMIXED* function in SAS. For instance *NLMIXED*, as used by some authors,^4,20^ tends to be fitted using the default dual quasi-Newton algorithm.^6^ Thus, for a symmetrical, positive definite matrix *B*^(*k*)^ which satisfies the secant condition, *B*^(*k*)^ is chosen so that it may be updated according to *B*^(*k*+1)^ = *B*^(*k*)^ + *A*^(*k*)^ (where *A*^(*k*)^ is a matrix which is easily estimated) whilst still preserving symmetry, positive definiteness and the secant condition. The Broyden, Fletcher, Goldfarb, and Shanno (BFGS) formula^21^ provides one approach where these conditions are satisfied and this is applied to the Cholesky factor of the approximate Hessian as the default method in the *NLMIXED* function.

For the purpose of comparison with the Newton–Raphson algorithms that follow, we focussed on *glmer* in R which is open source and readily available.^22^

## 4 Maximum likelihood estimations for bivariate model using NR algorithm

Here we demonstrate two different numerical methods for deriving maximum likelihood estimates (MLE) for the parameters in the bivariate random effects model used in test accuracy meta-analysis. They are both based on the Newton–Raphson (NR) algorithm,^7^ perhaps, one of the most common numerical methods used in optimisation. The NR algorithm is an iterative method for finding the roots of a differentiable function that generates a sequence of estimates which usually come increasingly close to the optimal solution. The algorithm is based on successive approximations to the solution, using Taylor's theorem to approximate the equation. It may be applied to both one-dimensional and higher dimensional problems by replacing the derivative with the gradient, and the reciprocal of the second derivative with the inverse of the Hessian matrix (see below).^23,24^

In essence, the task of maximum likelihood estimation may be reduced to a one of finding the roots to the derivatives of the log likelihood function, that is, finding $\alpha ,\beta ,\sigma_{A}^{2},\sigma_{B}^{2}\text{ and }\rho$ such that $\nabla l(\alpha ,\beta ,\sigma_{A}^{2},\sigma_{B}^{2},\rho )=0$. Hence, the NR algorithm may be used to solve this equation iteratively. Suppose that ${\hat{\theta }}_{k}={\left({\hat{\alpha }}_{k},\;{\hat{\beta }}_{k},\;{\hat{\sigma }}_{a}^{2}{\;}_{k},{\hat{\sigma }}_{b}^{2}{\;}_{k},\;{\hat{\rho }}_{k}\right)}^{T}$ is the *k*^th^ estimate of the vector of true parameters $\theta =(\alpha ,\beta ,\sigma_{a}^{2},\sigma_{b}^{2},\rho )T$ in the BRM with the log-likelihood function as given in equation (6). If we define the score statistic, S(θ^k), as the ∇l and the Hessian matrix, H(θ^k), such that

(10) $$S\left({\hat{\theta }}_{k}\right)={\left(\begin{array}{ccc} \frac{\partial l}{\partial {\hat{\alpha }}_{k}} & \frac{\partial l}{\partial {\hat{\beta }}_{k}} & \begin{array}{ccc} \frac{\partial l}{\partial {\hat{\sigma }}_{a}^{2}{\;}_{k}} & \frac{\partial l}{\partial {\hat{\sigma }}_{b}^{2}{\;}_{k}} & \frac{\partial l}{\partial {\hat{\rho }}_{k}} \end{array} \end{array}\right)}^{T}\;\;\;\;\;\;\;\;\;\;\;\;\;\;\;\;\;\;\;\;\;\;\;\;\;\;\;\;\;\;\;\;(10)$$

(11) H(θ^k)=(∂2l∂α^k2∂2l∂α^k∂β^k∂2l∂α^k∂σ^ak2∂2l∂α^k∂σ^bk2∂2l∂α^k∂ρ^k2∂2l∂β^k∂α^k∂2l∂β^k2∂2l∂β^k∂σ^ak2∂2l∂β^k∂σ^bk2∂2l∂β^k∂ρ^k∂2l∂σ^ak2∂α^k∂2l∂σ^ak2∂β^k∂2l∂σ^ak22∂2l∂σ^ak2∂σ^bk2∂2l∂σ^ak2∂ρ^k∂2l∂σ^bk2∂α^k∂2l∂σ^bk2∂β^k∂2l∂σ^bk2∂σ^ak2∂2l∂σ^bk22∂2l∂σ^bk2∂ρ^k∂2l∂ρ^k∂α^k∂2l∂ρ^k∂β^k∂2l∂ρ^k∂σ^ak2∂2l∂ρ^k∂σ^bk2∂2l∂ρ^k2)

then by using Taylor's expansion of the score function S(θ^k) we have

(12) S(θ^k+1)≈S(θ^k)+H(θ^k)(θ^k+1-θ^k)

Since S(θ^k+1)=0 when θ^k+1 maximises $l_{n}(\theta |x_{1},x_{2})$, we obtain the following estimate

(13) θ^k+1≈θ^k-H(θ^k)-1S(θ^k)

which is the *k*^th^ iteration of the Newton–Raphson algorithm based on the observed Fisher information (OFI) matrix (equivalent to the negative of the Hessian matrix) for estimating the five parameters in the BRM.

In order to calculate the derivatives in equations (10) and (11) numerically, one can use the simple approximation to the first order derivative in five dimensions with respect to the underlying estimated parameter. Suppose it is α^k, then the derivative can be approximated as

(14) $$\frac{\partial l}{\partial {\hat{\alpha }}_{k}}=\frac{f\left({\hat{\alpha }}_{k}+h,{\hat{\beta }}_{k},\;{\hat{\sigma }}_{a}^{2}{\;}_{k},{\hat{\sigma }}_{b}^{2}{\;}_{k},\;{\hat{\rho }}_{k}\right)-f\left({\hat{\theta }}_{k}{\;}^{T}\right)}{h}\;\;\;\;\;\;\;\;\;\;\;\;\;\;\;\;\;\;\;\;\;\;\;\;\;\;\;\;\;\;\;\;\;$$

or

(15) $$\frac{\partial l}{\partial {\hat{\alpha }}_{k}}=\frac{f\left({\hat{\alpha }}_{k}+h,{\hat{\beta }}_{k},\;{\hat{\sigma }}_{a}^{2}{\;}_{k},{\hat{\sigma }}_{b}^{2}{\;}_{k},\;{\hat{\rho }}_{k}\right)-f\left({\hat{\alpha }}_{k}-h,{\hat{\beta }}_{k},\;{\hat{\sigma }}_{a}^{2}{\;}_{k},{\hat{\sigma }}_{b}^{2}{\;}_{k},\;{\hat{\rho }}_{k}\right)}{2h}\;\;\;\;\;\;\;\;\;\;\;\;\;\;$$

where *h* is very small (h→0, for example h=0.0001), and ${\hat{\theta }}_{k}={\left({\hat{\alpha }}_{k},\;{\hat{\beta }}_{k},\;{\hat{\sigma }}_{a}^{2}{\;}_{k},{\hat{\sigma }}_{b}^{2}{\;}_{k},\;{\hat{\rho }}_{k}\right)}^{T}$. On the other hand, we can obtain a numerical approximation to the second-order derivative in five dimensions with respect to α^k using the formula

(16) $$\frac{\partial^{2}l}{\partial {\hat{\alpha }}_{k}{\;}^{2}}=\frac{f\left({\hat{\alpha }}_{k}+h,{\hat{\beta }}_{k},\;{\hat{\sigma }}_{a}^{2}{\;}_{k},{\hat{\sigma }}_{b}^{2}{\;}_{k},\;{\hat{\rho }}_{k}\right)-2f\left({\hat{\theta }}_{k}{\;}^{T}\right)+f({\hat{\alpha }}_{k}-h,{\hat{\beta }}_{k},\;{\hat{\sigma }}_{a}^{2}{\;}_{k},{\hat{\sigma }}_{b}^{2}{\;}_{k},\;{\hat{\rho }}_{k})}{h^{2}}\;\;\;\;\;\;$$

and the approximation to the second-order derivative in five dimensions with respect to α^k, β^k can be written as

(17) $$\frac{\partial^{2}l}{\partial {\hat{\alpha }}_{k}\partial {\hat{\beta }}_{k}}=\frac{f\left({\hat{\alpha }}_{k}+h,{\hat{\beta }}_{k}+h,\;{\hat{\sigma }}_{a}^{2}{\;}_{k},{\hat{\sigma }}_{b}^{2}{\;}_{k},\;{\hat{\rho }}_{k}\right)-2f\left({\hat{\theta }}_{k}{\;}^{T}\right)+f({\hat{\alpha }}_{k}-h,{\hat{\beta }}_{k}-h,\;{\hat{\sigma }}_{a}^{2}{\;}_{k},{\hat{\sigma }}_{b}^{2}{\;}_{k},\;{\hat{\rho }}_{k})}{2h^{2}-\left(\frac{\partial^{2}l}{\partial {\hat{\alpha }}_{k}{\;}^{2}}+\frac{\partial^{2}l}{\partial {\hat{\beta }}_{k}{\;}^{2}}\right)/2}\;\;$$

We can calculate the other elements in equations (10) and (11), in a similar fashion to those shown in equations (14) to (17). Alternatively one may use the ready-made functions in R, *grad* and *hessian*, in the package *numDeriv*.^25^

The double integration over the random effects in the log likelihood function in equation (6) is computed using the adaptive multidimensional integration algorithms described in Genz and Malik^26^ and Berntsen et al.^27^ It is written in C and may be accessed via the R wrapper *cubature*.^28^ We can use the function *adaptIntegrate* (within *cubature*) to perform adaptive multidimensional integration of vector-valued integrands over hypercubes, and get a solution to the integral in equation (6) and then estimate the five parameters in the BRM.

The first algorithm uses the profile of the log likelihood equation^6^ in equation (6) to estimate the five unknown parameters in equation (9) by starting with what may be called ‘robust initial values’. The robust initial values are starting values that are sufficiently close to the actual values of the parameters so they increase both the chances and the speed of convergence. The second algorithm is based on the observed Fisher information matrix^8^ where similar to the first algorithm, robust initial values provide the starting point to the algorithm before updating the observed Fisher information matrix.

## 5 The method of profiling

In order to explain the method of profiling,^8,29^ suppose that only two parameters α and β need to be estimated and that β^, the MLE for β, may be expressed as a function of α. The profile likelihood of α is then L(α,β^(α)) and is now a function of α only.^30^ If β^(α) is known explicitly, then maximising the profile likelihood with respect to α is achieved easily. However, when it is not known, β^(α) may be obtained numerically by fixing α and maximising *L*(α,β) with respect to β. Thus β^(α) takes a different value for each fixed value of α and α^ is the estimate for α which maximises the profile likelihood L(α,β^(α)). In practical terms, this means deriving profile likelihood estimates over a range of values for α and when there are more than two parameters to estimate, the range of values of the other parameters also need to be considered (see below).

Lindstrom and Bates^31^ pointed out that optimising the profile log-likelihood usually requires fewer iterations, the derivatives are somewhat simpler, and the convergence is more consistent. In addition, they have also encountered examples where the NR algorithm failed to converge when optimising the likelihood (which includes a variance term) but was able to optimise the profile likelihood with ease.

It is often difficult to determine whether an algorithm has converged upon a ‘local’ maximum instead of the ‘global’ maximum^32,33^ but many objective functions will have local maxima either due to the shape of the underlying function or due to noise introduced by the data. One approach to overcome this is to choose multiple initial values randomly and select the maximum these yield.^33^ Here a more systematic approach is taken, where the data from the studies help define a feasible space for the global maximum and an equally spaced grid is overlaid on the space.^34,35^ This is then used as the basis for a maximum likelihood approach in determining robust initial values. It represents the first phase of the algorithm. In the second phase, we update the estimations continuously, using the last estimated values, until we get the convergence.

The profile log likelihood algorithm for estimating the parameters in bivariate model:

5.1  Initial estimate phase: we can derive an initial estimate of the nuisance parameters ($\rho ,\sigma_{a}^{2},\sigma_{b}^{2}$) by following the profile log likelihood procedure outlined above. Specifically,
5.1a. Using the minimum and maximum of α and β across all the studies as bounds, and using the delta-method to estimate the range of $\sigma_{a}^{2},\sigma_{b}^{2}$, generate a regular equally-spaced sequence for each of $\sigma_{a}^{2},\sigma_{b}^{2},\alpha ,\beta$. Next, construct a grid of all possible combinations of values of ($\sigma_{a}^{2},\sigma_{b}^{2},\alpha ,\beta$) where each combination of $\left(\sigma_{a}^{2},\sigma_{b}^{2},\alpha ,\beta \right)$ generates a new log likelihood curve $l(\rho ,\sigma_{a}^{2}(\rho ),\sigma_{b}^{2}(\rho ),\alpha (\rho ),\beta (\rho ))$ over ρ. Choose the combination ($\sigma_{a,\mathrm{opt}1}^{2},\sigma_{b,\mathrm{opt}1}^{2},\alpha_{\mathrm{opt}1},\beta_{\mathrm{opt}1}$) which gives the largest likelihood over all these curves when ρ=0. The associated likelihood curve for this combination is then maximised with respect to ρ using the NR algorithm to give an initial estimate, ρ^0.
5.1b. Construct combinations of all the possible values of ($\sigma_{b}^{2},\alpha ,\beta$) as in 5.1a. Choose the combination of ($\sigma_{b,\mathrm{opt}2}^{2},\alpha_{\mathrm{opt}2},\beta_{\mathrm{opt}2}$) which gives the largest likelihood for ρ=ρ^0 and $\sigma_{a}^{2}=\sigma_{a,\mathrm{opt}1}^{2}$ from 5.1a. The associated likelihood curve for this combination with ρ=ρ^0 is maximised with respect to $\sigma_{a}^{2}$ using the NR algorithm to give an initial estimate ${\hat{\sigma }}_{a}^{2}{\;}_{0}$.
5.1c. As previously, construct combinations of all the possible values of (α,β) and choose the combination ($\alpha_{\mathrm{opt}3},\beta_{\mathrm{opt}3}$) which gives the largest likelihood for ρ=ρ^0, $\sigma_{a}^{2}={\hat{\sigma }}_{a}^{2}{\;}_{0}$ and $\sigma_{b}^{2}=\sigma_{b,opt2}^{2}$ is chosen. The associated likelihood curve for this combination with ρ=ρ^0 and $\sigma_{a}^{2}={\hat{\sigma }}_{a}^{2}{\;}_{0}$ is then maximised with respect to $\sigma_{b}^{2}$ using the NR algorithm to give an initial estimate σ^b02
5.1d. Following the same procedure, initial estimates for α^0 and β^0 may be derived.
5.2. The updating phase: based on the initial estimate ${\hat{\theta }}_{0}={\left({\hat{\alpha }}_{0},\;{\hat{\beta }}_{0},\;{\hat{\sigma }}_{a}^{2}{\;}_{0},{\hat{\sigma }}_{b}^{2}{\;}_{0},{\hat{\rho }}_{0}\right)}^{T}$ from 5.1, the algorithm iteratively updates each parameter separately with the other consecutive estimated parameters. In other words, the estimate ${\hat{\rho }}_{k}$ is updated with $\left({\hat{\alpha }}_{k},\;{\hat{\beta }}_{k},\;{\hat{\sigma }}_{a}^{2}{\;}_{k},{\hat{\sigma }}_{b}^{2}{\;}_{k}\right)$ to get ${\hat{\rho }}_{k+1}$ by maximising $l\left({\hat{\rho }}_{k},{\hat{\alpha }}_{k}({\hat{\rho }}_{k}),\;{\hat{\beta }}_{k}({\hat{\rho }}_{k}),\;{\hat{\sigma }}_{a}^{2}{\;}_{k}({\hat{\rho }}_{k}),{\hat{\sigma }}_{b}^{2}{\;}_{k}\left({\hat{\rho }}_{k}\right)\right)$ with respect to ${\hat{\rho }}_{k}$. Similarly, the estimate of ${\hat{\sigma }}_{a}^{2}{\;}_{k}$ is updated with $\left({\hat{\alpha }}_{k},\;{\hat{\beta }}_{k},{\hat{\sigma }}_{b}^{2}{\;}_{k},{\hat{\rho }}_{k+1}\right)$ to get ${\hat{\sigma }}_{a}^{2}{\;}_{k+1}$, ${\hat{\sigma }}_{b}^{2}{\;}_{k}$ with $\left({\hat{\alpha }}_{k},\;{\hat{\beta }}_{k},{\hat{\sigma }}_{a}^{2}{\;}_{k+1},{\hat{\rho }}_{k+1}\right)$ to get ${\hat{\sigma }}_{b}^{2}{\;}_{k+1}$, ${\hat{\alpha }}_{k}$ with $\left({\hat{\beta }}_{k},{\hat{\sigma }}_{a}^{2}{\;}_{k+1},{\hat{\sigma }}_{b}^{2}{\;}_{k+1},{\hat{\rho }}_{k+1}\right)$ to get ${\hat{\alpha }}_{k+1}$, and ${\hat{\beta }}_{k}$ with $\left({\hat{\alpha }}_{k+1},{\hat{\sigma }}_{a}^{2}{\;}_{k+1},{\hat{\sigma }}_{b}^{2}{\;}_{k+1},{\hat{\rho }}_{k+1}\right)$ to get ${\hat{\beta }}_{k+1}$. So, at the end of this process we have ${\hat{\theta }}_{k+1}\;=\;({\hat{\alpha }}_{k+1},{\hat{\beta }}_{k+1},{\hat{\sigma }}_{a}^{2}{\;}_{k+1},{\hat{\sigma }}_{b}^{2}{\;}_{k+1},{\hat{\rho }}_{k+1})$.
5.3. While |θ^k+1-θ^k|>ɛ, set k=k+1 and repeat 5.2 until convergence is achieved.

Although the algorithm is straightforward, compared with the observed Fisher information algorithm below, it is more computationally expensive and is likely to be more time consuming as a result. In particular, the second phase involves several iterations, as the NR algorithm is applied to each of the five parameters individually in each update until convergence is achieved. Moreover, the log likelihood function is evaluated over many different possible combinations of the parameters' values.

## 6 Observed Fisher information with robust initial values (OFIRIV)

Although the method of profiling circumvents the local maximum problem by generating robust initial parameter values, it is computationally expensive. In contrast, the observed Fisher information is more efficient than the method of profiling but without appropriate starting values there is still the risk of it converging on a local maximum.

Here the approach of ascertaining robust initial parameter values is combined with an algorithm based on the observed Fisher information.^8^ This has the potential of improving on the previous algorithm by increasing the computational efficiency.

Thus, the algorithm is as follows:

6.1.  Initial estimate phase: get an initial estimate ${\hat{\theta }}_{0}={\left({\hat{\alpha }}_{0},\;{\hat{\beta }}_{0},\;{\hat{\sigma }}_{a}^{2}{\;}_{0},{\hat{\sigma }}_{b}^{2}{\;}_{0},{\hat{\rho }}_{0}\right)}^{T}$ for the parameters ($\alpha ,\beta ,\sigma_{a}^{2},\sigma_{b}^{2},\rho$) by using the algorithm described in 5.1a to 5.1d
6.2.   Updating phase: the next steps use the observed Fisher information matrix^8^ to update the estimates for the parameters in the BRM.
6.2a. Let $\theta ={\left(\alpha ,\;\beta ,\sigma_{a}^{2},\sigma_{b}^{2},\rho \;\right)}^{T}$ be the vector of parameters to be estimated in the BRM with log-likelihood function defined in equation (6), set k=0 and choose the initial value ${\hat{\theta }}_{0}={\left({\hat{\alpha }}_{0},\;{\hat{\beta }}_{0},\;{\hat{\sigma }}_{a}^{2}{\;}_{0},{\hat{\sigma }}_{b}^{2}{\;}_{0},\;{\hat{\rho }}_{0}\right)}^{T}$ from 6.1 to start the algorithm.
6.2b. Calculate the score statistic S(θ^k) and the Hessian matrix H(θ^k) as in equations (10) and (11), respectively.
6.2c.  Estimate θ^k+1 based on θ^k such that: θ^k+1=θ^k-[H(θ^k)]-1 S(θ^k).
6.2d. Check whether θ^k+1 is optimal using the convergence condition |θ^k+1-θ^k|≤ɛ, where ɛ expresses the desired tolerance level and is usually very small, for example ɛ = 10^-12^.
6.2e. While |θ^k+1-θ^k|>ɛ, set k=k+1 and repeat 6.2b to 6.2d until we get convergence.

To ensure stability of the algorithm, we may control for jumps in individual components of the parameter vector between iterations and redirect the algorithm to the robust initial value for the component. For example, if the difference |α^k+1-α^k| between successive iterations is too large, then we may reset α^k+1 to α^0.

Other criteria may be used for terminating the iteration. Recall that obtaining the maximum likelihood estimate is equivalent to finding the roots to the score statistic S(θ^k), then a suitable stopping criterion would be when |S(θ^k)2≤ɛ|. Alternatively we may use -[H(θ^k)]-1 S(θ^k)2≤ɛ – asymptotically the observed Fisher information is equivalent to the variance of the score statistic, so this criterion has the advantage of being insensitive to scaling of the variables. Occasionally, a parameter estimate may recur, so that θ^k is exactly equal to θ^k+m for *m* > 1. At this point, the algorithm has entered a limit cycle and a stopping rule is required so that it does not continue indefinitely.

Compared to the profile log likelihood algorithm, this algorithm consumes less time than the former and is computationally more straightforward. Furthermore, once the Hessian matrix has been estimated at the initial step, (H(θ^0)), this may be used for subsequent iterations thereby saving computation time. However, if the Hessian matrix is estimated at each iteration then the algorithm will converge after fewer iterations than if H(θ^0) is used throughout, but will nonetheless take longer on average and the risk of getting a singular matrix will be much higher which leads to a lower convergence rate. Here H(θ^0) was used as the estimate of the Hessian for each iteration.

It is well known that the choice of initial values can be important in the speed of convergence, the ability of the algorithm to find a global maximum, and the ability to converge at all.^36,^ ^37^ However, specifically for Newton–Raphson-based methods, Kantorovich's theorem provides the theoretical underpinning for the importance of the choice of initial values and the success of convergence.^38^ Essentially around the start point, the behaviour of the Jacobian of the function and its inverse have to meet certain conditions on continuity and boundedness if the algorithm is to converge.

Here we applied a grid across a bounded space for the parameters^29^ before taking a maximum likelihood approach to generate robust initial values for the parameters. However, there is no guarantee the algorithm with robust initial values will produce parameter estimates that uniquely maximises the log-likelihood. Whilst the choice of robust initial values may lower the risk of the algorithm converging on a local maximum,^39^ it cannot eliminate this risk. Essentially identifying the global maximum is still a heuristic process no matter what initial values are chosen.

Furthermore when the data are noisy, rather than converging on a local maximum, the algorithms may fail to converge at all. Generally, this occurs when one or more elements in the score function or Hessian returns an infinity, the absolute value of the correlation exceeds 1, or a negative variance begins to emerge. To cope with these types of situation, we may reset the variable responsible to either the value in a previous iteration or to the initial value. If this occurs in the initial value estimate phase, the resetting of the variable may involve setting the value on the grid that maximises the likelihood. If the correlation is the problem variable in the initial estimate phase, the Pearson correlation coefficient for the observed data may be used. These measures allow the algorithm to proceed on a slightly modified trajectory. Both algorithms discussed in this and the preceding section accommodate these scenarios in this way.

An alternative approach for obtaining the MLEs of the parameters is to transform all or part of the model in order to facilitate convergence. This is used by the two generic packages as discussed in section 3.

## 7 Numerical examples

In this section, the two algorithms are evaluated through a simulated study before applying them to two real case examples. In each case, they are compared with the *glmer* function from the package *lme4* in R,^5^ which has been previously validated. All analyses were conducted in R^22^ and the code for each of the algorithms appears in the online appendices.

### 7.1 Simulation study

For the simulation study, the true values of the five parameters were set to: α=1.2; β=2.5; $\sigma_{a}^{2}=0.4$; $\sigma_{b}^{2}=0.6$; and ρ=-0.7. The number of studies *k* included in the meta-analysis was set at 10 and 20. Thus, the logit sensitivity $\alpha_{i}$ and logit specificity $\beta_{i}$ for the *i*^th^ study were simulated from

(18) $$\begin{array}{c} (\begin{array}{c} \alpha_{i}\\ \beta_{i} \end{array})∼N((\begin{array}{c} 1.2\\ 2.5 \end{array}),(\begin{array}{cc} 0.4 & -0.3429\\ -0.3429 & 0.6 \end{array})) \end{array}$$

This provides the study-specific sensitivity, $P_{A,i}=\mathrm{logi}t^{-1}(\alpha_{i})$ and specificity $P_{B,i}=\mathrm{logi}t^{-1}(\beta_{i})$. For each study *i*, the number of non-diseased $n_{B,i}$ was generated randomly to be between 10 and 200 and the diseased $n_{A,i}$, chosen to be 0.25$n_{B,i}$ rounded to the nearest whole number. Thus, for each of *k* studies, the true positives $TP_{i}$, and true negatives $TN_{i}$ were simulated from the binomial distributions detailed in equations (3) and (4).

For each of the three algorithms (including *glmer*) the BRM was applied to 10,000 simulated data sets of size *k* = 10 and then *k* = 20. The results were compared using the convergence rate, mean squared error (MSE), average relative error (ARE), mean bias and coverage probability.

Table 1 gives the convergence rates (CR) for each of the three algorithms. It is clear that the *glmer* function does not converge for all the datasets achieving at most 84% for *k = *20 studies. This contrasts the profile likelihood and OFIRIV methods which both have near 100% convergence. Also increasing the number of studies improves convergence for all the methods.

**Table 1.** The convergence rates calculated from 10,000 simulations for each method at *k* = 10 and 20.

	Convergence Rate	
Method	k = 10	k = 20
*glmer*	0.8026	0.8365
Profile likelihood	1.0000	1.0000
OFIRIV	0.9888	0.9951

As heterogeneity is one of the factors contributing to non-convergence, restricting the analysis to the converged data sets potentially may make the overall sample less heterogeneous. Thus, we may expect the mean square errors (MSE) and average relative error (ARE) to be lower for the *glmer* function, where the converged set was 15% smaller than the other two algorithms. This is observed in Tables 2 and 3 below, although the differences are small.

**Table 2.** MSE of the estimated values of the five parameters for the different methods at *k* = 10 and 20 based on converged samples from 10,000 simulations. The values in bold refer to the lowest MSE between all the methods.

Method	*k* = 10					*k* = 20				
	α^	β^	σ^a2	σ^b2	ρ^	α^	β^	σ^a2	σ^b2	ρ^
*glmer*	**0.0727**	0.0859	**0.1020**	**0.1395**	0.1834	**0.0343**	**0.0409**	**0.0479**	**0.0668**	0.0660
Profile	0.0778	**0.0840**	0.1090	0.1516	0.1190	0.0359	0.0432	0.1013	0.0944	**0.0465**
OFIRIV	0.0808	0.1133	0.2620	0.1530	**0.1087**	0.0421	0.0596	0.1059	0.1537	0.0542

**Table 3.** ARE of the estimated values of the five parameters for the different methods at *k* = 10 and 20 based on converged samples from 10,000 simulations.

Method	*k* = 10					*k* = 20				
	α^	β^	σ^a2	σ^b2	ρ^	α^	β^	σ^a2	σ^b2	ρ^
*glmer*	**0.1784**	0.0929	0.5956	**0.4847**	0.4427	**0.1227**	**0.0640**	**0.4350**	**0.3447**	0.2879
Profile	0.1792	**0.0917**	**0.5924**	0.4854	**0.3163**	0.1253	0.0656	0.5041	0.3529	**0.2212**
OFIRIV	0.1848	0.0963	0.6475	0.4975	0.3167	0.1342	0.0698	0.5675	0.3816	0.2347

Note: The values in bold refer to the ARE between all the methods.

The mean bias of the estimated values of the five parameters for each of the four methods is given in Table 4. Similar to the previous tables, the results are comparable across the different methods with no one method giving a consistently better performance over all five parameters.

**Table 4.** Mean bias of the estimated values of the five parameters for the different methods at *k* = 10 and 20 based on converged samples from 10,000 simulations.

Method	*k* = 10					*k* = 20				
	α^	β^	σ^a2	σ^b2	ρ^	α^	β^	σ^a2	σ^b2	ρ^
*glmer*	**0.0106**	−0.0114	−0.0127	**−0.0518**	**0.0001**	**0.0027**	−0.0089	−0.0339	−0.0445	**−0.0257**
Profile	0.0107	0.0051	**−0.0107**	−0.0604	0.0842	0.0098	0.0041	**0.0311**	**−0.0235**	0.0330
OFIRIV	0.0108	**0.0045**	0.0158	−0.0573	0.1006	0.0094	**0.0018**	0.0658	−0.0032	0.0524

Note: The values in bold refer to the lowest absolute bias between all the methods.

Table 5 shows the coverage probabilities of the confidence ellipses for (α,β) as estimated using methods previously described.^40^ The method of profiling produces the highest coverage probability for both cases.

**Table 5.** The coverage probability of the 95% confidence regions for (α,β) based on the converged samples from 10,000 simulations for each method at *k* = 10 and 20.

	Coverage probability	
Method	*k* = 10	*k* = 20
*glmer*	0.9442	0.9359
Profile likelihood	0.9483	0.9396
OFIRIV	0.9457	0.9395

It is clear that the different methods are comparable across a number of statistics. However, the *glmer* function does have a substantially lower convergence rates than the other two algorithms. Thus based on its superior convergence rate and coverage probability, the profile likelihood is recommended as the method of choice for estimating the parameters for the bivariate random effects model in meta-analysis.

To illustrate the contrasting performance, three examples where *glmer* failed to converge are compared with the profile likelihood and the OFIRIV algorithms which did converge. The three simulated data sets are based on 10 studies and may be found in the online Appendix. For the first example, *glmer*'s failure to converge was due to it calculating an inconsistent gradient value in some iterations (max|grad| = 0.0105486). For this example, the profile likelihood estimates of (α^, β^, σ^a2, σ^b2, ρ^) converged after five iterations to (1.2696185, 2.3511021, 0.4482178, 0.4121124, −0.4127191) and the OFIRIV converged after nine iterations to (1.3075654, 2.3844844, 0.5144346, 0.4182635, −0.4127191).

In the second example, *glmer* returned a NAN for the correlation coefficient and two warning messages. The first was that it was unable to evaluate a scaled gradient and the second that there was a degenerate Hessian matrix with negative eigenvalues. The profile likelihood estimates of (α^, β^, σ^a2, σ^b2, ρ^) converged after four iterations to (1.6202820, 2.3936771, 0.1321239, 0.5772051, −0.1337343) and the OFIRIV algorithm converged after six iterations to (1.6266015, 2.3164244, 0.1321239, 0.5618353, −0.1624175).

In the third example, *glmer* failed to converge due to producing a correlation coefficient ρ^=-1 which makes equation (9) undefined. Furthermore, the algorithm gave an inconsistent gradient value for some iterations (max|grad| = 0.00106003). In contrast, the profile likelihood estimates of (α^, β^, σ^a2, σ^b2, ρ^) converged after nine iterations to (1.0629164, 2.5540033, 0.3010341, 0.6466069, −0.7733131) and the OFIRIV algorithm converged after 962 iterations to (1.18282870, 2.59699272, 0.03534702, 0.19803168, −0.77331306).

### 7.2 Real data examples

In this section, the three algorithms described are applied to two previously published test accuracy reviews.^41,42^ For each of these reviews, the five parameters in the BRM in equation (6) were estimated by the three algorithms and their performances compared.

#### 7.2.1 Computed tomography of the distant metastasis

The first review evaluated the accuracy of several imaging modalities in detecting cancer including 98 studies published between 1990 and 2009.^41^ Here the focus will be on the accuracy of computed tomography (CT) in identifying distant metastases where there were 12 relevant studies. The data may be found in the supplementary materials of Chen et al.^13^

In Table 6, the estimates of the five parameters in logit space for each of the algorithms are given for the CT data. The number of iterations required to achieve convergence by each algorithm is also given. In general, the estimated values produced from profile likelihood and the OFIRIV algorithms are very close to those estimated by the *glmer* function.

**Table 6.** The estimation results (in logit space) based on the different algorithms for the CT dataset.

Algorithm	α^	β^	σ^a2	σ^b2	ρ^	Iterations
Profile log likelihood	0.6254442	1.8819420	0.2793882	0.1736081	−0.7742788	10
OFIRIV	0.6254440	1.8819419	0.2793883	0.1736061	−0.7742770	15
*glmer*	0.6256095	1.8821715	0.2766782	0.1728707	−0.778121	205

Note: For glmer this is the number of iterations of the Nelder–Mead algorithm.

As point of illustration, Tables 7 and 8 give the successive estimates for α, β, $\sigma_{a}^{2},\sigma_{b}^{2}$ and ρ for the profile log likelihood and OFIRIV algorithms at each iteration. As may be seen from both tables, the robust initial values for the profile likelihood and the OFIRIV are within a close proximity of the final estimates for the parameters. This enables more rapid convergence and reduces the risk of converging on a local maximum. Convergence is achieved after 10 iterations for the profile likelihood algorithm and 15 iterations for the OFIRIV algorithm. In general, *glmer* requires a greater number of iterations before the convergence conditions are satisfied.

**Table 7.** Estimates for α, β, $\sigma_{a}^{2},\sigma_{b}^{2}$ and ρ (in logit space) at each iteration for the profile log likelihood algorithm for the CT dataset.

Iteration	α^	β^	σ^a2	σ^b2	ρ^
RIV	0.5799135	1.8898890	0.2555894	0.2135463	−0.5987675
1	0.6158106	1.8817761	0.2708390	0.1809543	−0.7216847
2	0.6237156	1.8815193	0.2767653	0.1752273	−0.7605525
3	0.6237154	1.8822370	0.2788309	0.1739427	−0.7708735
4	0.6251286	1.8819709	0.2789662	0.1737936	−0.7731979
5	0.6253686	1.8819368	0.2792944	0.1736791	−0.7738299
6	0.6253686	1.8819368	0.2793657	0.1736244	−0.7741466
7	0.6254275	1.8819371	0.2793582	0.1736228	−0.7741466
8	0.6254413	1.8819399	0.2793822	0.1736115	−0.7742438
9	0.6254440	1.8819415	0.2793869	0.1736086	−0.7742722
10	0.6254442	1.8819420	0.2793882	0.1736081	−0.7742788

Note: RIV are the robust initial values that enter the updating part of the algorithm.

**Table 8.** Estimates for α, β, $\sigma_{a}^{2},\sigma_{b}^{2}$ and ρ (in logit space) at each iteration for the OFIRIV algorithm for CT data. RIV are the robust initial values that enter the updating part of the algorithm.

iteration	α^	β^	σ^a2	σ^b2	ρ^
RIV	0.5799135	1.8898890	0.2555894	0.2135463	−0.5987675
1	0.6235215	1.8828534	0.2694258	0.1723672	−0.7705554
2	0.6243623	1.8819306	0.2779604	0.1743782	−0.7730370
3	0.6252492	1.8819956	0.2790256	0.1733277	−0.7736512
4	0.6254016	1.8819652	0.2793110	0.1738137	−0.7744848
5	0.6254305	1.8819280	0.2793691	0.1734812	−0.7740802
6	0.6254439	1.8819544	0.2793845	0.1736935	−0.7743981
7	0.6254419	1.8819342	0.2793871	0.1735523	−0.7741988
8	0.6254449	1.8819475	0.2793882	0.1736450	−0.7743325
9	0.6254433	1.8819386	0.2793882	0.1735839	−0.7742451
10	0.6254445	1.8819445	0.2793884	0.1736241	−0.7743028
11	0.6254437	1.8819406	0.2793883	0.1735976	−0.7742648
12	0.6254442	1.8819432	0.2793883	0.1736150	−0.7742898
13	0.6254439	1.8819415	0.2793883	0.1736035	−0.7742733
14	0.6254441	1.8819426	0.2793883	0.1736111	−0.7742842
15	0.6254440	1.8819419	0.2793883	0.1736061	−0.7742770

**Table 9.** The estimation results (in logit space) based on the different algorithms for the PHQ-9 dataset.^42^

Algorithm	α^	β^	σ^a2	σ^b2	ρ^	Iterations
Profile log likelihood	1.0575056	2.3793688	0.4784003	0.6340357	−0.5801280	7
OFIRIV	1.0575050	2.3793687	0.4784016	0.6340387	−0.5801333	57
*glmer*	1.057142	2.379097	0.4742536	0.6313224	−0.5837202	212

Note: For *glmer* this is the number of iterations of the Nelder–Mead algorithm.

Also of note is the behaviour of each algorithm which shows smooth changes between iterations without any wild fluctuations. This is because the algorithms start with robust initial values that are sufficiently close to the real value of the parameters thereby increasing the stability of the algorithms.

#### 7.2.2 Screening for depression based on the PHQ-9

The second dataset used is a review which evaluated the accuracy of the Patient Health Questionnaire (PHQ-9) in screening for depression. The PHQ-9 consists of nine questions and is a recognised screening tool for depression. Willis and Hyde^42^ conducted a meta-analysis which evaluated its accuracy and the data used here may be found in the supplemental appendix.^42^ There were 10 included studies.

For each algorithm, Table 6 gives the estimated values of the five parameters for the PHQ-9 data and the number of iterations needed for convergence. Like the previous example, the OFIRIV algorithm and profile log likelihood algorithm give results that are close to those from the *glmer* function. Although the OFIRIV executes more iterations than the profile likelihood before convergence is attained, it still executes far fewer than the *glmer* function.

## 8 Discussion

Meta-analysis is integral to evidence synthesis providing a means of summarising research from multiple primary studies. Its widespread uptake has coincided with developments in the meta-analysis methods used, progressing from fixed effects methods^43^ to including study-specific random effects,^44^ and from univariate outcomes^44^ to using multivariate outcomes.^45^

This has increased the complexity of the type of models used and the optimisation methods needed to estimate the unknown parameters. The most common model used in test accuracy meta-analyses is a bivariate generalised linear mixed model, and is often referred to as the bivariate random effects model (BRM). The complexity of this model lies with the need to perform a double integration over the random effects and an integrand which is a binomial-normal mixture distribution. Having no closed form, numerical methods are required to estimate the parameters of interest. Although generic functions such as *glmer* in the *lme4* package in R^5^ and *NLMIXED* in SAS^6^ may be used to fit the BRM, they remain ‘black boxes’ to the vast majority of users.

Here we have demonstrated from first principles how maximum likelihood estimates may be derived using Newton–Raphson-based approaches to provide estimates for the parameters of interest in the BRM used in test accuracy meta-analyses. In this respect, the proposed algorithms appear to have received little attention in the literature.

Both the method of profiling and the Observed Fisher Information matrix algorithm perform well and give accurate estimates for the five unknown parameters of the BRM. However, without suitable modifications, they still have the potential to breakdown either by converging on biased estimates, the so-called ‘local maxima problem’,^39^ or not converge at all.

One way to address the local maxima problem is to choose the initial values for the parameters more carefully. Here we get robust initial values by first using the data to derive a grid across a feasible space of values for the parameters. Then each parameter is estimated independently based on values of the other parameters that maximise the log likelihood function with respect to the parameter being estimated. This method is aimed at providing initial values which are close to the true values for the parameters to increase the chances of converging on these true values.

The second issue is that the algorithm may fail to converge at all, particularly when there are noisy data. There may be a number of reasons for this, including difficulty in calculating the partial second derivatives in the Hessian matrix due to their being a very small rate of change or that an inverse for the Hessian matrix may not exist. The correlation may become out of bounds or one or more of the variances may take on negative values. Essentially this represents a recurring challenge for multi-parameter models – how to ensure the optimisation algorithm reliably converges on an accurate estimate.

To deal with this, some authors advocate transforming the model to an alternative parameterisation such as those used by the generic packages discussed earlier. For example, the model may be transformed so that the covariance matrix or Hessian matrix remains positive definite throughout successive iterations. Whilst this offers a substantial improvement, for the *glmer* function at least, it does not lead to convergence in all cases. This was clearly demonstrated by the simulation study.

Another approach is to monitor the iterative process for aberrant parameter estimates or function values and reset to a value from a previous iteration when this occurs. For example, when a parameter estimate strays out of the space of feasible values, or a derivative becomes infinite. This recognises there may be many trajectories that converge on a stable estimate and resetting the current estimate of a parameter may move the algorithm onto a different trajectory. This was the method used in both the profile likelihood and the OFIRIV algorithms and the convergence rates were 100% and close to 100%, respectively.

Both algorithms developed in this study perform better than the *glmer* function in terms of convergence and coverage probability whilst being comparable in other performance characteristics such as mean squared error, mean bias and average relative error. However, due to its superior convergence rate and coverage probability, we recommend the method of profiling over the OFIRIV.

Furthermore the OFIRIV and method of profile algorithms benefit from having been developed specifically to estimate the parameters in the BRM, in contrast to the *glmer* function which is designed to fit a range of different models. Perhaps this indicates that as the models get more sophisticated, algorithms which are specifically optimised for the task may become more important.

Other Newton–Raphson-based approaches are possible, such as the method of scoring which uses the expected Fisher information matrix.^46^ In principle, this method should improve the stability of the algorithm by ensuring the Hessian matrix is positive definite. However, for the BRM it involves two integrations, one over the random effects and the other to estimate the expectation of the Hessian matrix and technically this is not straight forward as well as being computationally time-consuming.

Although the focus here has been on developing algorithms which estimated the sensitivity and specificity in a BRM, the same approach could easily be extended to estimating parameters when study-level covariates are included in the BRM. Such meta-regression analyses are common place when investigating heterogeneity between studies and may improve the potential validity of any estimates.^47^ Equally the algorithms could be applied to recently developed tailored models which augment the applicability of test accuracy research by combining meta-analyses with routine data.^48,49^

The study does have some limitations. Although the OFIRIV and method of profiling algorithms demonstrate high performance characteristics and compare favourably with the one of the generic functions in R, a more extensive investigation is required to firmly establish their utility and limitations. This would involve evaluating them over a greater variety of cases, including examples with sparse data.^50^

Many of the functions used to fit the BRM invoke generic optimisation methods^5,6^ that are used to fit other models. For example, *glmer* uses Nelder–Mead^18^ and BOBYQA^19^ and *NLMIXED* uses a dual quasi-Newton algorithm^6^ as the default algorithm across all types of models. One of the conclusions which may be drawn from this study is that it may be for the BRM a more specific optimisation approach would overcome some of the convergence issues that have been previously reported in other studies.^50^ This could be investigated using simulated examples over a range of optimisation algorithms.

The emphasis here has been to be explicit in the methods used to fit the bivariate random effects model and demonstrate how this may be done from first principles using the open source programming language R.^22^ However, as an interpretative language, R is slow for such models and the code may take several minutes to run. The computational time could be significantly improved by translating the algorithms into a low-level compiled language such as C.

In summary, we have developed two algorithms based on Newton–Raphson methods to fit specifically the bivariate random effects model used in meta-analysis of test accuracy studies. From a simulation study, it was demonstrated that both algorithms had higher convergence rates and coverage probability than those from the *glmer* function whilst having similar performance characteristics in other measures. Overall the profile likelihood approach had the best performance characteristics for fitting the bivariate random effects model out of the three methods. Future research should focus on improving the computational time of these algorithms.

## Supplemental Material

Supplemental Material1 - Supplemental material for Maximum likelihood estimation based on Newton–Raphson iteration for the bivariate random effects model in test accuracy meta-analysis Click here for additional data file. Supplemental material, Supplemental Material1 for Maximum likelihood estimation based on Newton–Raphson iteration for the bivariate random effects model in test accuracy meta-analysis by Brian H Willis, Mohammed Baragilly and Dyuti Coomar in Statistical Methods in Medical Research

Supplemental Material2 - Supplemental material for Maximum likelihood estimation based on Newton–Raphson iteration for the bivariate random effects model in test accuracy meta-analysis Click here for additional data file. Supplemental material, Supplemental Material2 for Maximum likelihood estimation based on Newton–Raphson iteration for the bivariate random effects model in test accuracy meta-analysis by Brian H Willis, Mohammed Baragilly and Dyuti Coomar in Statistical Methods in Medical Research
