# Supplementary material for: Maximum likelihood estimation based on Newton–Raphson iteration for the bivariate random effects model in test accuracy meta-analysis
Source: Stat Methods Med Res. 2019 Jun 11;29(4):1197–211. doi: 10.1177/0962280219853602 (PMC7221455; doi:10.1177/0962280219853602)
Supplement: Supplemental Material1 - Supplemental material for Maximum likelihood estimation based on Newton–Raphson iteration for the bivariate random effects model in test accuracy meta-analysis [file Supplemental_Material1.pdf]

Appendix 1: R-code for method of profiling:

```
#Example: PHQ-9 data:
######################
id = c(1,2,3,4,5,6,7,8,9,10)
TP = c(121,4,18,24,86,33,14,4,43,27)
FN<-c(42,2,12,6,89,3,5,3,7,4)
FP<-c(220,2,29,26,59,13,38,2,90,21)
TN<-c(2259,145,121,301,1117,47,222,95,1392,94)
na<-TP+FN
nb<-TN+FP
mydata = data.frame(cbind(id,na,TP,nb,TN,FN,FP))
names(mydata) = c("id", "na", "TP", "nb", "TN", "FN", "FP")
k=nrow(mydata)
Sen<-TP/na
for (i in 1:k){if(na[i]==0) Sen[i]<-0}
Spe<-TN/nb
for (i in 1:k){if(nb[i]==0) Spe[i]<-0}
mydata$Sen <-Sen
mydata$Spe <-Spe

library(cubature)

dbivlognorm <- function(pa,pb,alpha, beta, sig2a, sig2b, rho){
  (2*pi*sqrt(sig2a*sig2b*(1-rho^2))*(pa*(1-pa)*pb*(1-pb)))^(-1)*exp(-1/(2*(1-
rho^2))*
  ((qlogis(pa)-alpha)^2/sig2a + (qlogis(pb)-beta)^2/sig2b - (2*rho*(qlogis(pa)-
alpha)*(qlogis(pb)-beta))/sqrt(sig2a*sig2b)))
}

integrand <- function(p,ya,na,yb,nb,alpha, beta, sig2a, sig2b, rho){
  pa = p[1]
  pb = p[2]
  dbinom(ya, size=na, prob=pa)*dbinom(yb, size=nb,
prob=pb)*dbivlognorm(pa,pb,alpha,beta,sig2a,sig2b,rho)
}

f4<-function(rho) {
  int <- rep(NA, length=k)
  for(i in 1:k){
    int[i] <- adaptIntegrate(integrand, lowerLimit = c(0, 0), upperLimit = c(1,
1),ya=mydata$TP[i],na=mydata$na[i],yb=mydata$TN[i],nb=mydata$nb[i],
alpha=alpha.ini, beta=beta.ini, sig2a=sig2a.ini, sig2b=sig2b.ini, rho)$integral
  }
  return(sum(log(int)))
}

r.log.Sen <- range(qlogis(Sen), finite = TRUE)
r.log.Spe <- range(qlogis(Spe), finite = TRUE)
logit.Sen.var <- 1/(na*Sen*(1-Sen))
r.log.Sen.var <- range(logit.Sen.var, finite = TRUE)
logit.Spe.var <- 1/(nb*Spe*(1-Spe))
r.log.Spe.var <- range(logit.Spe.var, finite = TRUE)
x1 <- seq(r.log.Sen[1],r.log.Sen[2], length.out = 5)
x2 <- seq(r.log.Spe[1],r.log.Spe[2], length.out = 5)
x3 <- seq(r.log.Sen.var[1], r.log.Sen.var[2], length.out = 5)
x4 <- seq(r.log.Spe.var[1], r.log.Spe.var[2], length.out = 5)
alpha.beta.sig2a.sig2b <- expand.grid(x1,x2,x3,x4)
alpha.beta.sig2a.sig2b <- as.matrix(alpha.beta.sig2a.sig2b)
log.lik <- NULL
for (i in 1:dim(alpha.beta.sig2a.sig2b)[1]){
   alpha.ini=alpha.beta.sig2a.sig2b[i,][1]
   beta.ini=alpha.beta.sig2a.sig2b[i,][2]
   sig2a.ini=alpha.beta.sig2a.sig2b[i,][3]
   sig2b.ini=alpha.beta.sig2a.sig2b[i,][4]
   log.lik <- c(log.lik, f4(0))
```

```
}

alpha.ini <- alpha.beta.sig2a.sig2b[which.max(log.lik),][1]
beta.ini <- alpha.beta.sig2a.sig2b[which.max(log.lik),][2]
sig2a.ini <- alpha.beta.sig2a.sig2b[which.max(log.lik),][3]
sig2b.ini <- alpha.beta.sig2a.sig2b[which.max(log.lik),][4]
cor.Se.Sp<-cor(Sen, Spe, method = c("pearson"))

mle.rho <- function(f4, rho0=0, N=20, tol=1E-5) {
        h <- 0.0000001
        i <- 1; rho1 <- rho0
        p4 <- numeric(N)
        while (i<=N) {
                if(rho0 <= -1 | rho0 >= 1) {
                  p4[i] <- cor.Se.Sp
                  i<- i + 1
                  break
                }
                fh <- f4(rho0+h)
                f0 <- f4(rho0)
                fhm <- f4(rho0 - h)
                fd <- (fh-f0)/h
                sd <- (fh-2*f0+ fhm)/h^2
                if(sd == 0 | sd == -Inf | fd == -Inf | (fd/sd)==-Inf | (fd/sd)==Inf
| is.na(fd)==TRUE  | is.na(sd)==TRUE) {
                  p4[i]=cor.Se.Sp
                  i<- i + 1
                  break
                }
                rho1 <- (rho0 - (fd/sd))
                p4[i] <- rho1
                i <- i + 1
                if (abs(rho1-rho0) < tol) break
                rho0 <- rho1
        }
        return(p4[1:(i-1)])
}

p4 <- mle.rho(f4)
rho.est<-p4[length(p4)]
rho.est

f2<-function(sig2a) {
  int <- rep(NA, length=k)
  for(i in 1:k){
    int[i] <- adaptIntegrate(integrand, lowerLimit = c(0, 0), upperLimit = c(1,
1),ya=mydata$TP[i],na=mydata$na[i],yb=mydata$TN[i],nb=mydata$nb[i],
alpha=alpha.ini, beta=beta.ini, sig2a, sig2b=sig2b.ini, rho=rho.est)$integral
  }
  return(sum(log(int)))
}
x1 <- seq(r.log.Sen[1],r.log.Sen[2], length.out = 10)
x2 <- seq(r.log.Spe[1],r.log.Spe[2], length.out = 10)
x4 <- seq(r.log.Spe.var[1], r.log.Spe.var[2], length.out = 10)
alpha.beta.sig2b <- expand.grid(x1,x2,x4)
alpha.beta.sig2b <- as.matrix(alpha.beta.sig2b)
log.lik <- NULL
for (i in 1:dim(alpha.beta.sig2b)[1]){
   alpha.ini=alpha.beta.sig2b[i,][1]
   beta.ini=alpha.beta.sig2b[i,][2]
   sig2b.ini=alpha.beta.sig2b[i,][3]
   log.lik <- c(log.lik, f2(sig2a.ini))
}
alpha.ini <- alpha.beta.sig2b[which.max(log.lik),][1]
beta.ini <- alpha.beta.sig2b[which.max(log.lik),][2]
sig2b.ini <- alpha.beta.sig2b[which.max(log.lik),][3]

mle.sig2a <- function(f2, sig2a0=sig2a.ini, N=20, tol=1E-5) {
```

```
        h <- 0.0000001
        i <- 1; sig2a1 <- sig2a0
        p2 <- numeric(N)
        while (i<=N) {
                if(sig2a0 <= 0) {
                  p2[i] <- sig2a.ini
                  i<- i + 1
                  break
                }
                fh <- f2(sig2a0+h)
                f0 <- f2(sig2a0)
                fhm <- f2(sig2a0 - h)
                fd <- (fh-f0)/h
                sd <- (fh-2*f0+ fhm)/h^2
                if(sd == 0 | sd == -Inf | fd == -Inf | (fd/sd)==-Inf | (fd/sd)==Inf
| is.na(fd)==TRUE  | is.na(sd)==TRUE) {
                  p2[i]=sig2a.ini
                  i<- i + 1
                  break
                }
                sig2a1 <- (sig2a0 - (fd/sd))
                p2[i] <- sig2a1
                i <- i + 1
                if (abs(sig2a1-sig2a0) < tol) break
                sig2a0 <- sig2a1
        }
        return(p2[1:(i-1)])
}

p2 <- mle.sig2a(f2)
sig2a.est<-p2[length(p2)]
sig2a.est

f3<-function(sig2b) {
  int <- rep(NA, length=k)
  for(i in 1:k){
    int[i] <- adaptIntegrate(integrand, lowerLimit = c(0, 0), upperLimit = c(1,
1),ya=mydata$TP[i],na=mydata$na[i],yb=mydata$TN[i],nb=mydata$nb[i],
alpha=alpha.ini, beta=beta.ini, sig2a=sig2a.est, sig2b, rho=rho.est)$integral
  }
  return(sum(log(int)))
}
x1 <- seq(r.log.Sen[1],r.log.Sen[2], length.out = 10)
x2 <- seq(r.log.Spe[1],r.log.Spe[2], length.out = 10)
alpha.beta <- expand.grid(x1,x2)
alpha.beta <- as.matrix(alpha.beta)
log.lik <- NULL
for (i in 1:dim(alpha.beta)[1]){
   alpha.ini=alpha.beta[i,][1]
   beta.ini=alpha.beta[i,][2]
   log.lik <- c(log.lik, f3(sig2b.ini))
}
alpha.ini <- alpha.beta[which.max(log.lik),][1]
beta.ini <- alpha.beta[which.max(log.lik),][2]

mle.sig2b <- function(f3, sig2b0=sig2b.ini, N=20, tol=1E-5) {
        h <- 0.0000001
        i <- 1; sig2b1 <- sig2b0
        p3 <- numeric(N)
        while (i<=N) {
                if(sig2b0 <= 0) {
                  p3[i] <- sig2b.ini
                  i<- i + 1
                  break
                }
                fh <- f3(sig2b0+h)
                f0 <- f3(sig2b0)
                fhm <- f3(sig2b0 - h)
```

```
                fd <- (fh-f0)/h
                sd <- (fh-2*f0+ fhm)/h^2
                if(sd == 0 | sd == -Inf | fd == -Inf | (fd/sd)==-Inf | (fd/sd)==Inf
| is.na(fd)==TRUE  | is.na(sd)==TRUE) {
                  p3[i]=sig2b.ini
                  i<- i + 1
                  break
                }
                sig2b1 <- (sig2b0 - (fd/sd))
                p3[i] <- sig2b1
                i <- i + 1
                if (abs(sig2b1-sig2b0) < tol) break
                sig2b0 <- sig2b1
        }
        return(p3[1:(i-1)])
}

p3 <- mle.sig2b(f3)
sig2b.est<-p3[length(p3)]
sig2b.est

f<-function(alpha) {
  int <- rep(NA, length=k)
  for(i in 1:k){
    int[i] <- adaptIntegrate(integrand, lowerLimit = c(0, 0), upperLimit = c(1,
1),ya=mydata$TP[i],na=mydata$na[i],yb=mydata$TN[i],nb=mydata$nb[i], alpha,
beta=beta.ini, sig2a=sig2a.est, sig2b=sig2b.est, rho=rho.est)$integral
  }
  return(sum(log(int)))
}

mle.alpha <- function(f, alpha0=alpha.ini, N=20, tol=1E-5) {
        h <- 0.0000001
        i <- 1; alpha1 <- alpha0
        p <- numeric(N)
        while (i<=N) {
                fh <- f(alpha0+h)
                f0 <- f(alpha0)
                fhm <- f(alpha0 - h)
                fd <- (fh-f0)/h
                sd <- (fh-2*f0+ fhm)/h^2
                if(sd == 0 | sd == -Inf | fd == -Inf | (fd/sd)==-Inf | (fd/sd)==Inf
| is.na(fd)==TRUE  | is.na(sd)==TRUE) {
                  p[i]=alpha.ini
                  i<- i + 1
                  break
                }
                alpha1 <- (alpha0 - (fd/sd))
                p[i] <- alpha1
                i <- i + 1
                if (abs(alpha1-alpha0) < tol) break
                alpha0 <- alpha1
        }
        return(p[1:(i-1)])
}
p <- mle.alpha(f)
alpha.est<-p[length(p)]
alpha.est

f1<-function(beta) {
  int <- rep(NA, length=k)
  for(i in 1:k){
    int[i] <- adaptIntegrate(integrand, lowerLimit = c(0, 0), upperLimit = c(1,
1),ya=mydata$TP[i],na=mydata$na[i],yb=mydata$TN[i],nb=mydata$nb[i],
alpha=alpha.est, beta, sig2a=sig2a.est, sig2b=sig2b.est, rho=rho.est)$integral
  }
  return(sum(log(int)))
}
```

```
mle.beta <- function(f1, beta0=beta.ini, N=20, tol=1E-5) {
        h <- 0.0000001
        i <- 1; beta1 <- beta0
        p1 <- numeric(N)
        while (i<=N) {
                fh <- f1(beta0+h)
                f0 <- f1(beta0)
                fhm <- f1(beta0 - h)
                fd <- (fh-f0)/h
                sd <- (fh-2*f0+ fhm)/h^2
                if(sd == 0 | sd == -Inf | fd == -Inf | (fd/sd)==-Inf | (fd/sd)==Inf
| is.na(fd)==TRUE  | is.na(sd)==TRUE) {
                  p1[i]=beta.ini
                  i<- i + 1
                  break
                }
                beta1 <- (beta0 - (fd/sd))
                p1[i] <- beta1
                i <- i + 1
                if (abs(beta1-beta0) < tol) break
                beta0 <- beta1
        }
        return(p1[1:(i-1)])
}
p1 <- mle.beta(f1)
beta.est<-p1[length(p1)]
beta.est

i <- 1
N<-10000
tolerance <- 0.001
est <- rbind(numeric(N),numeric(N),numeric(N),numeric(N),numeric(N))
count <- 0

while (i<=N) {
  if (count > 0) tolerance <- 0.00001
  f4<-function(rho) {
    int <- rep(NA, length=k)
    for(i in 1:k){
      int[i] <- adaptIntegrate(integrand, lowerLimit = c(0, 0), upperLimit = c(1,
1),ya=mydata$TP[i],na=mydata$na[i],yb=mydata$TN[i],nb=mydata$nb[i],
alpha=alpha.est, beta=beta.est, sig2a=sig2a.est, sig2b=sig2b.est, rho)$integral
    }
    return(sum(log(int)))
  }
  mle.rho <- function(f4, rho0=rho.est, N=20, tol=tolerance) {
          h <- 0.0000001
          i <- 1; rho1 <- rho0
          p4 <- numeric(N)
          while (i<=N) {
                  if(rho0 <= -1 | rho0 >= 1) {
                    p4[i] <- rho.est
                    i<- i + 1
                    break
                  }
                  fh <- f4(rho0+h)
                  f0 <- f4(rho0)
                  fhm <- f4(rho0 - h)
                  fd <- (fh-f0)/h
                  sd <- (fh-2*f0+ fhm)/h^2
                  if(sd == 0 | sd == -Inf | fd == -Inf | (fd/sd)==-Inf |
(fd/sd)==Inf | is.na(fd)==TRUE  | is.na(sd)==TRUE) {
                    p4[i]=rho.est
                    i<- i + 1
                    break
                  }
                  rho1 <- (rho0 - (fd/sd))
```

```
                p4[i] <- rho1
                i <- i + 1
                if (abs(rho1-rho0) < tol) break
                rho0 <- rho1
        }
        return(p4[1:(i-1)])
  }
  p4 <- mle.rho(f4)
  rho.est1<-p4[length(p4)]

  f2<-function(sig2a) {
    int <- rep(NA, length=k)
    for(i in 1:k){
      int[i] <- adaptIntegrate(integrand, lowerLimit = c(0, 0), upperLimit = c(1,
1),ya=mydata$TP[i],na=mydata$na[i],yb=mydata$TN[i],nb=mydata$nb[i],
alpha=alpha.est, beta=beta.est, sig2a, sig2b=sig2b.est, rho=rho.est1)$integral
    }
    return(sum(log(int)))
  }
  mle.sig2a <- function(f2, sig2a0=sig2a.est, N=20, tol=tolerance) {
        h <- 0.0000001
        i <- 1; sig2a1 <- sig2a0
        p2 <- numeric(N)
        while (i<=N) {
                if(sig2a0 <= 0) {
                  p2[i] <- sig2a.est
                  i<- i + 1
                  break
                }
                fh <- f2(sig2a0+h)
                f0 <- f2(sig2a0)
                fhm <- f2(sig2a0 - h)
                fd <- (fh-f0)/h
                sd <- (fh-2*f0+ fhm)/h^2
                if(sd == 0 | sd == -Inf | fd == -Inf | (fd/sd)==-Inf |
(fd/sd)==Inf | is.na(fd)==TRUE  | is.na(sd)==TRUE) {
                  p2[i]=sig2a.est
                  i<- i + 1
                  break
                }
                sig2a1 <- (sig2a0 - (fd/sd))
                p2[i] <- sig2a1
                i <- i + 1
                if (abs(sig2a1-sig2a0) < tol) break
                sig2a0 <- sig2a1
        }
        return(p2[1:(i-1)])
  }
  p2 <- mle.sig2a(f2)
  sig2a.est1<-p2[length(p2)]

  f3<-function(sig2b) {
    int <- rep(NA, length=k)
    for(i in 1:k){
      int[i] <- adaptIntegrate(integrand, lowerLimit = c(0, 0), upperLimit = c(1,
1),ya=mydata$TP[i],na=mydata$na[i],yb=mydata$TN[i],nb=mydata$nb[i],
alpha=alpha.est, beta=beta.est, sig2a=sig2a.est1, sig2b, rho=rho.est1)$integral
    }
    return(sum(log(int)))
  }
  mle.sig2b <- function(f3, sig2b0=sig2b.est, N=20, tol=tolerance) {
        h <- 0.0000001
        i <- 1; sig2b1 <- sig2b0
        p3 <- numeric(N)
        while (i<=N) {
                if(sig2b0 <= 0) {
                  p3[i] <- sig2b.est
                  i<- i + 1
```

```
                    break
                  }
                  fh <- f3(sig2b0+h)
                  f0 <- f3(sig2b0)
                  fhm <- f3(sig2b0 - h)
                  fd <- (fh-f0)/h
                  sd <- (fh-2*f0+ fhm)/h^2
                  if(sd == 0 | sd == -Inf | fd == -Inf | (fd/sd)==-Inf |
(fd/sd)==Inf | is.na(fd)==TRUE  | is.na(sd)==TRUE) {
                    p3[i]=sig2b.est
                    i<- i + 1
                    break
                  }
                  sig2b1 <- (sig2b0 - (fd/sd))
                  p3[i] <- sig2b1
                  i <- i + 1
                  if (abs(sig2b1-sig2b0) < tol) break
                  sig2b0 <- sig2b1
          }
          return(p3[1:(i-1)])
  }
  p3 <- mle.sig2b(f3)
  sig2b.est1<-p3[length(p3)]

  f<-function(alpha) {
    int <- rep(NA, length=k)
    for(i in 1:k){
      int[i] <- adaptIntegrate(integrand, lowerLimit = c(0, 0), upperLimit = c(1,
1),ya=mydata$TP[i],na=mydata$na[i],yb=mydata$TN[i],nb=mydata$nb[i], alpha,
beta=beta.est, sig2a=sig2a.est1, sig2b=sig2b.est1, rho=rho.est1)$integral
    }
    return(sum(log(int)))
  }
  mle.alpha <- function(f, alpha0=alpha.est, N=20, tol=tolerance) {
          h <- 0.0000001
          i <- 1; alpha1 <- alpha0
          p <- numeric(N)
          while (i<=N) {
                  fh <- f(alpha0+h)
                  f0 <- f(alpha0)
                  fhm <- f(alpha0 - h)
                  fd <- (fh-f0)/h
                  sd <- (fh-2*f0+ fhm)/h^2
                  if(sd == 0 | sd == -Inf | fd == -Inf | (fd/sd)==-Inf |
(fd/sd)==Inf | is.na(fd)==TRUE  | is.na(sd)==TRUE) {
                    p[i]=alpha.est
                    i<- i + 1
                    break
                  }
                  alpha1 <- (alpha0 - (fd/sd))
                  p[i] <- alpha1
                  i <- i + 1
                  if (abs(alpha1-alpha0) < tol) break
                  alpha0 <- alpha1
          }
          return(p[1:(i-1)])
  }
  p <- mle.alpha(f)
  alpha.est1<-p[length(p)]

  f1<-function(beta) {
    int <- rep(NA, length=k)
    for(i in 1:k){
      int[i] <- adaptIntegrate(integrand, lowerLimit = c(0, 0), upperLimit = c(1,
1),ya=mydata$TP[i],na=mydata$na[i],yb=mydata$TN[i],nb=mydata$nb[i],
alpha=alpha.est1, beta, sig2a=sig2a.est1, sig2b=sig2b.est1, rho=rho.est1)$integral
    }
    return(sum(log(int)))
```

```
  }
  mle.beta <- function(f1, beta0=beta.est, N=20, tol=tolerance) {
          h <- 0.0000001
          i <- 1; beta1 <- beta0
          p1 <- numeric(N)
          while (i<=N) {
                  fh <- f1(beta0+h)
                  f0 <- f1(beta0)
                  fhm <- f1(beta0 - h)
                  fd <- (fh-f0)/h
                  sd <- (fh-2*f0+ fhm)/h^2
                  if(sd == 0 | sd == -Inf | fd == -Inf | (fd/sd)==-Inf |
(fd/sd)==Inf | is.na(fd)==TRUE  | is.na(sd)==TRUE) {
                    p1[i]=beta.est
                    i<- i + 1
                    break
                  }
                  beta1 <- (beta0 - (fd/sd))
                  p1[i] <- beta1
                  i <- i + 1
                  if (abs(beta1-beta0) < tol) break
                  beta0 <- beta1
          }
          return(p1[1:(i-1)])
  }
  p1 <- mle.beta(f1)
  beta.est1<-p1[length(p1)]
  est[,i] <- rbind(alpha.est1, beta.est1, sig2a.est1, sig2b.est1, rho.est1)
  i <- i + 1
  if (abs(alpha.est1-alpha.est) < tolerance && abs(beta.est1-beta.est) < tolerance
&& abs(sig2a.est1-sig2a.est) < tolerance && abs(sig2b.est1-sig2b.est) < tolerance
&& abs(rho.est1-rho.est) < tolerance) break
  alpha.est <- alpha.est1; beta.est<-beta.est1; sig2a.est<-sig2a.est1; sig2b.est<-
sig2b.est1; rho.est<-rho.est1
  count <-1
}
est[,1:(i-1)]
profile.est<-est[,(i-1)]
profile.est
```

Appendix 2: R-code for OFIRIV

```
#Example: PHQ-9 data:
######################
id = c(1,2,3,4,5,6,7,8,9,10)
TP = c(121,4,18,24,86,33,14,4,43,27)
FN<-c(42,2,12,6,89,3,5,3,7,4)
FP<-c(220,2,29,26,59,13,38,2,90,21)
TN<-c(2259,145,121,301,1117,47,222,95,1392,94)
na<-TP+FN
nb<-TN+FP
mydata = data.frame(cbind(id,na,TP,nb,TN,FN,FP))
names(mydata) = c("id", "na", "TP", "nb", "TN", "FN", "FP")
k=nrow(mydata)
Sen<-TP/na
for (i in 1:k){if(na[i]==0) Sen[i]<-0}
Spe<-TN/nb
for (i in 1:k){if(nb[i]==0) Spe[i]<-0}
mydata$Sen <-Sen
mydata$Spe <-Spe

library(cubature)

dbivlognorm <- function(pa,pb,alpha, beta, sig2a, sig2b, rho){
  (2*pi*sqrt(sig2a*sig2b*(1-rho^2))*(pa*(1-pa)*pb*(1-pb)))^(-1)*exp(-1/(2*(1-
rho^2))*
  ((qlogis(pa)-alpha)^2/sig2a + (qlogis(pb)-beta)^2/sig2b - (2*rho*(qlogis(pa)-
alpha)*(qlogis(pb)-beta))/sqrt(sig2a*sig2b)))
}

integrand <- function(p,ya,na,yb,nb,alpha, beta, sig2a, sig2b, rho){
  pa = p[1]
  pb = p[2]
  dbinom(ya, size=na, prob=pa)*dbinom(yb, size=nb,
prob=pb)*dbivlognorm(pa,pb,alpha,beta,sig2a,sig2b,rho)
}

f4<-function(rho) {
  int <- rep(NA, length=k)
  for(i in 1:k){
    int[i] <- adaptIntegrate(integrand, lowerLimit = c(0, 0), upperLimit = c(1,
1),ya=mydata$TP[i],na=mydata$na[i],yb=mydata$TN[i],nb=mydata$nb[i],
alpha=alpha.ini, beta=beta.ini, sig2a=sig2a.ini, sig2b=sig2b.ini, rho)$integral
  }
  return(sum(log(int)))
}

r.log.Sen <- range(qlogis(Sen), finite = TRUE)
r.log.Spe <- range(qlogis(Spe), finite = TRUE)
logit.Sen.var <- 1/(na*Sen*(1-Sen))
r.log.Sen.var <- range(logit.Sen.var, finite = TRUE)
logit.Spe.var <- 1/(nb*Spe*(1-Spe))
r.log.Spe.var <- range(logit.Spe.var, finite = TRUE)
x1 <- seq(r.log.Sen[1],r.log.Sen[2], length.out = 5)
x2 <- seq(r.log.Spe[1],r.log.Spe[2], length.out = 5)
x3 <- seq(r.log.Sen.var[1], r.log.Sen.var[2], length.out = 5)
x4 <- seq(r.log.Spe.var[1], r.log.Spe.var[2], length.out = 5)
alpha.beta.sig2a.sig2b <- expand.grid(x1,x2,x3,x4)
alpha.beta.sig2a.sig2b <- as.matrix(alpha.beta.sig2a.sig2b)
log.lik <- NULL
for (i in 1:dim(alpha.beta.sig2a.sig2b)[1]){
   alpha.ini=alpha.beta.sig2a.sig2b[i,][1]
   beta.ini=alpha.beta.sig2a.sig2b[i,][2]
   sig2a.ini=alpha.beta.sig2a.sig2b[i,][3]
   sig2b.ini=alpha.beta.sig2a.sig2b[i,][4]
   log.lik <- c(log.lik, f4(0))
}
```

```
alpha.ini <- alpha.beta.sig2a.sig2b[which.max(log.lik),][1]
beta.ini <- alpha.beta.sig2a.sig2b[which.max(log.lik),][2]
sig2a.ini <- alpha.beta.sig2a.sig2b[which.max(log.lik),][3]
sig2b.ini <- alpha.beta.sig2a.sig2b[which.max(log.lik),][4]
cor.Se.Sp<-cor(Sen, Spe, method = c("pearson"))

mle.rho <- function(f4, rho0=0, N=20, tol=1E-5) {
        h <- 0.0000001
        i <- 1; rho1 <- rho0
        p4 <- numeric(N)
        while (i<=N) {
                if(rho0 <= -1 | rho0 >= 1) {
                  p4[i] <- cor.Se.Sp
                  i<- i + 1
                  break
                }
                fh <- f4(rho0+h)
                f0 <- f4(rho0)
                fhm <- f4(rho0 - h)
                fd <- (fh-f0)/h
                sd <- (fh-2*f0+ fhm)/h^2
                if(sd == 0 | sd == -Inf | fd == -Inf | (fd/sd)==-Inf | (fd/sd)==Inf
| is.na(fd)==TRUE  | is.na(sd)==TRUE) {
                  p4[i]=cor.Se.Sp
                  i<- i + 1
                  break
                }
                rho1 <- (rho0 - (fd/sd))
                p4[i] <- rho1
                i <- i + 1
                if (abs(rho1-rho0) < tol) break
                rho0 <- rho1
        }
        return(p4[1:(i-1)])
}

p4 <- mle.rho(f4)
rho.est<-p4[length(p4)]
rho.est

f2<-function(sig2a) {
  int <- rep(NA, length=k)
  for(i in 1:k){
    int[i] <- adaptIntegrate(integrand, lowerLimit = c(0, 0), upperLimit = c(1,
1),ya=mydata$TP[i],na=mydata$na[i],yb=mydata$TN[i],nb=mydata$nb[i],
alpha=alpha.ini, beta=beta.ini, sig2a, sig2b=sig2b.ini, rho=rho.est)$integral
  }
  return(sum(log(int)))
}
x1 <- seq(r.log.Sen[1],r.log.Sen[2], length.out = 10)
x2 <- seq(r.log.Spe[1],r.log.Spe[2], length.out = 10)
x4 <- seq(r.log.Spe.var[1], r.log.Spe.var[2], length.out = 10)
alpha.beta.sig2b <- expand.grid(x1,x2,x4)
alpha.beta.sig2b <- as.matrix(alpha.beta.sig2b)
log.lik <- NULL
for (i in 1:dim(alpha.beta.sig2b)[1]){
   alpha.ini=alpha.beta.sig2b[i,][1]
   beta.ini=alpha.beta.sig2b[i,][2]
   sig2b.ini=alpha.beta.sig2b[i,][3]
   log.lik <- c(log.lik, f2(sig2a.ini))
}
alpha.ini <- alpha.beta.sig2b[which.max(log.lik),][1]
beta.ini <- alpha.beta.sig2b[which.max(log.lik),][2]
sig2b.ini <- alpha.beta.sig2b[which.max(log.lik),][3]

mle.sig2a <- function(f2, sig2a0=sig2a.ini, N=20, tol=1E-5) {
        h <- 0.0000001
```

```
        i <- 1; sig2a1 <- sig2a0
        p2 <- numeric(N)
        while (i<=N) {
                if(sig2a0 <= 0) {
                  p2[i] <- sig2a.ini
                  i<- i + 1
                  break
                }
                fh <- f2(sig2a0+h)
                f0 <- f2(sig2a0)
                fhm <- f2(sig2a0 - h)
                fd <- (fh-f0)/h
                sd <- (fh-2*f0+ fhm)/h^2
                if(sd == 0 | sd == -Inf | fd == -Inf | (fd/sd)==-Inf | (fd/sd)==Inf
| is.na(fd)==TRUE  | is.na(sd)==TRUE) {
                  p2[i]=sig2a.ini
                  i<- i + 1
                  break
                }
                sig2a1 <- (sig2a0 - (fd/sd))
                p2[i] <- sig2a1
                i <- i + 1
                if (abs(sig2a1-sig2a0) < tol) break
                sig2a0 <- sig2a1
        }
        return(p2[1:(i-1)])
}

p2 <- mle.sig2a(f2)
sig2a.est<-p2[length(p2)]
sig2a.est

f3<-function(sig2b) {
  int <- rep(NA, length=k)
  for(i in 1:k){
    int[i] <- adaptIntegrate(integrand, lowerLimit = c(0, 0), upperLimit = c(1,
1),ya=mydata$TP[i],na=mydata$na[i],yb=mydata$TN[i],nb=mydata$nb[i],
alpha=alpha.ini, beta=beta.ini, sig2a=sig2a.est, sig2b, rho=rho.est)$integral
  }
  return(sum(log(int)))
}
x1 <- seq(r.log.Sen[1],r.log.Sen[2], length.out = 10)
x2 <- seq(r.log.Spe[1],r.log.Spe[2], length.out = 10)
alpha.beta <- expand.grid(x1,x2)
alpha.beta <- as.matrix(alpha.beta)
log.lik <- NULL
for (i in 1:dim(alpha.beta)[1]){
   alpha.ini=alpha.beta[i,][1]
   beta.ini=alpha.beta[i,][2]
   log.lik <- c(log.lik, f3(sig2b.ini))
}
alpha.ini <- alpha.beta[which.max(log.lik),][1]
beta.ini <- alpha.beta[which.max(log.lik),][2]

mle.sig2b <- function(f3, sig2b0=sig2b.ini, N=20, tol=1E-5) {
        h <- 0.0000001
        i <- 1; sig2b1 <- sig2b0
        p3 <- numeric(N)
        while (i<=N) {
                if(sig2b0 <= 0) {
                  p3[i] <- sig2b.ini
                  i<- i + 1
                  break
                }
                fh <- f3(sig2b0+h)
                f0 <- f3(sig2b0)
                fhm <- f3(sig2b0 - h)
                fd <- (fh-f0)/h
```

```
                sd <- (fh-2*f0+ fhm)/h^2
                if(sd == 0 | sd == -Inf | fd == -Inf | (fd/sd)==-Inf | (fd/sd)==Inf
| is.na(fd)==TRUE  | is.na(sd)==TRUE) {
                  p3[i]=sig2b.ini
                  i<- i + 1
                  break
                }
                sig2b1 <- (sig2b0 - (fd/sd))
                p3[i] <- sig2b1
                i <- i + 1
                if (abs(sig2b1-sig2b0) < tol) break
                sig2b0 <- sig2b1
        }
        return(p3[1:(i-1)])
}

p3 <- mle.sig2b(f3)
sig2b.est<-p3[length(p3)]
sig2b.est

f<-function(alpha) {
  int <- rep(NA, length=k)
  for(i in 1:k){
    int[i] <- adaptIntegrate(integrand, lowerLimit = c(0, 0), upperLimit = c(1,
1),ya=mydata$TP[i],na=mydata$na[i],yb=mydata$TN[i],nb=mydata$nb[i], alpha,
beta=beta.ini, sig2a=sig2a.est, sig2b=sig2b.est, rho=rho.est)$integral
  }
  return(sum(log(int)))
}

mle.alpha <- function(f, alpha0=alpha.ini, N=20, tol=1E-5) {
        h <- 0.0000001
        i <- 1; alpha1 <- alpha0
        p <- numeric(N)
        while (i<=N) {
                fh <- f(alpha0+h)
                f0 <- f(alpha0)
                fhm <- f(alpha0 - h)
                fd <- (fh-f0)/h
                sd <- (fh-2*f0+ fhm)/h^2
                if(sd == 0 | sd == -Inf | fd == -Inf | (fd/sd)==-Inf | (fd/sd)==Inf
| is.na(fd)==TRUE  | is.na(sd)==TRUE) {
                  p[i]=alpha.ini
                  i<- i + 1
                  break
                }
                alpha1 <- (alpha0 - (fd/sd))
                p[i] <- alpha1
                i <- i + 1
                if (abs(alpha1-alpha0) < tol) break
                alpha0 <- alpha1
        }
        return(p[1:(i-1)])
}
p <- mle.alpha(f)
alpha.est<-p[length(p)]
alpha.est

f1<-function(beta) {
  int <- rep(NA, length=k)
  for(i in 1:k){
    int[i] <- adaptIntegrate(integrand, lowerLimit = c(0, 0), upperLimit = c(1,
1),ya=mydata$TP[i],na=mydata$na[i],yb=mydata$TN[i],nb=mydata$nb[i],
alpha=alpha.est, beta, sig2a=sig2a.est, sig2b=sig2b.est, rho=rho.est)$integral
  }
  return(sum(log(int)))
}
```

```
mle.beta <- function(f1, beta0=beta.ini, N=20, tol=1E-5) {
        h <- 0.0000001
        i <- 1; beta1 <- beta0
        p1 <- numeric(N)
        while (i<=N) {
                fh <- f1(beta0+h)
                f0 <- f1(beta0)
                fhm <- f1(beta0 - h)
                fd <- (fh-f0)/h
                sd <- (fh-2*f0+ fhm)/h^2
                if(sd == 0 | sd == -Inf | fd == -Inf | (fd/sd)==-Inf | (fd/sd)==Inf
| is.na(fd)==TRUE  | is.na(sd)==TRUE) {
                  p1[i]=beta.ini
                  i<- i + 1
                  break
                }
                beta1 <- (beta0 - (fd/sd))
                p1[i] <- beta1
                i <- i + 1
                if (abs(beta1-beta0) < tol) break
                beta0 <- beta1
        }
        return(p1[1:(i-1)])
}
p1 <- mle.beta(f1)
beta.est<-p1[length(p1)]
beta.est

theta.start = c(alpha.est, beta.est, sig2a.est, sig2b.est, rho.est)

y<-function(theta) {
  alpha<- theta[1]
  beta<- theta[2]
  sig2a<- theta[3]
  sig2b<- theta[4]
  rho<- theta[5]
  int <- rep(NA, length=k)
  for(i in 1:k){
    int[i] <- adaptIntegrate(integrand, lowerLimit = c(0, 0), upperLimit = c(1,
1),ya=mydata$TP[i],na=mydata$na[i],yb=mydata$TN[i],nb=mydata$nb[i], alpha, beta,
sig2a, sig2b, rho)$integral
  }
  return(sum(log(int)))
}

OFI <- function(mydata,y, theta0 = theta.start, N=10000, tol=1E-5) {
        i <- 1; theta1 <- theta0
        p <- rbind(numeric(N),numeric(N),numeric(N),numeric(N),numeric(N))
        h <- 0.0000001
        f<-function(alpha) {
          int <- rep(NA, length=k)
          for(i in 1:k){
            int[i] <- adaptIntegrate(integrand, lowerLimit = c(0, 0), upperLimit =
c(1, 1),ya=mydata$TP[i],na=mydata$na[i],yb=mydata$TN[i],nb=mydata$nb[i], alpha,
beta=theta0[2], sig2a=theta0[3], sig2b=theta0[4], rho=theta0[5])$integral
          }
          return(sum(log(int)))
        }
        f1<-function(beta) {
          int <- rep(NA, length=k)
          for(i in 1:k){
            int[i] <- adaptIntegrate(integrand, lowerLimit = c(0, 0), upperLimit =
c(1, 1),ya=mydata$TP[i],na=mydata$na[i],yb=mydata$TN[i],nb=mydata$nb[i],
alpha=theta0[1], beta, sig2a=theta0[3], sig2b=theta0[4], rho=theta0[5])$integral
          }
          return(sum(log(int)))
        }
        f2<-function(sig2a) {
```

```
          int <- rep(NA, length=k)
          for(i in 1:k){
            int[i] <- adaptIntegrate(integrand, lowerLimit = c(0, 0), upperLimit =
c(1, 1),ya=mydata$TP[i],na=mydata$na[i],yb=mydata$TN[i],nb=mydata$nb[i],
alpha=theta0[1], beta=theta0[2], sig2a, sig2b=theta0[4], rho=theta0[5])$integral
          }
          return(sum(log(int)))
        }
        f3<-function(sig2b) {
          int <- rep(NA, length=k)
          for(i in 1:k){
            int[i] <- adaptIntegrate(integrand, lowerLimit = c(0, 0), upperLimit =
c(1, 1),ya=mydata$TP[i],na=mydata$na[i],yb=mydata$TN[i],nb=mydata$nb[i],
alpha=theta0[1], beta=theta0[2], sig2a=theta0[3], sig2b, rho=theta0[5])$integral
          }
          return(sum(log(int)))
        }
        f4<-function(rho) {
          int <- rep(NA, length=k)
          for(i in 1:k){
            int[i] <- adaptIntegrate(integrand, lowerLimit = c(0, 0), upperLimit =
c(1, 1),ya=mydata$TP[i],na=mydata$na[i],yb=mydata$TN[i],nb=mydata$nb[i],
alpha=theta0[1], beta=theta0[2], sig2a=theta0[3], sig2b=theta0[4], rho)$integral
          }
          return(sum(log(int)))
        }
        sd.alpha <- (y(c(theta0[1]+h, theta0[2],theta0[3],theta0[4],theta0[5]))-
2*y(theta0)+ y(c(theta0[1]-h, theta0[2],theta0[3],theta0[4],theta0[5]))))/h^2
        sd.beta <- (y(c(theta0[1], theta0[2]+h,theta0[3],theta0[4],theta0[5]))-
2*y(theta0)+ y(c(theta0[1], theta0[2]-h,theta0[3],theta0[4],theta0[5]))))/h^2
        sd.sig2a <- (y(c(theta0[1], theta0[2],theta0[3]+h,theta0[4],theta0[5]))-
2*y(theta0)+ y(c(theta0[1], theta0[2],theta0[3]-h,theta0[4],theta0[5]))))/h^2
        sd.sig2b <- (y(c(theta0[1], theta0[2],theta0[3],theta0[4]+h,theta0[5]))-
2*y(theta0)+ y(c(theta0[1], theta0[2],theta0[3],theta0[4]-h,theta0[5]))))/h^2
        sd.rho <- (y(c(theta0[1], theta0[2],theta0[3],theta0[4],theta0[5]+h))-
2*y(theta0)+ y(c(theta0[1], theta0[2],theta0[3],theta0[4],theta0[5]-h)))/h^2
        sd.alpha.beta <- (y(c(theta0[1]+h,
theta0[2]+h,theta0[3],theta0[4],theta0[5]))-2*y(theta0) + y(c(theta0[1]-h,
theta0[2]-h,theta0[3],theta0[4],theta0[5]))))/(2*h^2) - ((sd.alpha + sd.beta)/2)
        sd.alpha.sig2a <- (y(c(theta0[1]+h,
theta0[2],theta0[3]+h,theta0[4],theta0[5]))-2*y(theta0) + y(c(theta0[1]-h,
theta0[2],theta0[3]-h,theta0[4],theta0[5]))))/(2*h^2) - ((sd.alpha + sd.sig2a)/2)
        sd.alpha.sig2b <- (y(c(theta0[1]+h,
theta0[2],theta0[3],theta0[4]+h,theta0[5]))-2*y(theta0) + y(c(theta0[1]-h,
theta0[2],theta0[3],theta0[4]-h,theta0[5]))))/(2*h^2) - ((sd.alpha + sd.sig2b)/2)
        sd.alpha.rho <- (y(c(theta0[1]+h,
theta0[2],theta0[3],theta0[4],theta0[5]+h))-2*y(theta0) + y(c(theta0[1]-h,
theta0[2],theta0[3],theta0[4],theta0[5]-h)))/(2*h^2) - ((sd.alpha + sd.rho)/2)
        sd.beta.sig2a <- (y(c(theta0[1],
theta0[2]+h,theta0[3]+h,theta0[4],theta0[5]))-2*y(theta0) + y(c(theta0[1],
theta0[2]-h,theta0[3]-h,theta0[4],theta0[5]))))/(2*h^2) - ((sd.beta + sd.sig2a)/2)
        sd.beta.sig2b <- (y(c(theta0[1],
theta0[2]+h,theta0[3],theta0[4]+h,theta0[5]))-2*y(theta0) + y(c(theta0[1],
theta0[2]-h,theta0[3],theta0[4]-h,theta0[5]))))/(2*h^2) - ((sd.beta + sd.sig2b)/2)
        sd.beta.rho <- (y(c(theta0[1],
theta0[2]+h,theta0[3],theta0[4],theta0[5]+h))-2*y(theta0) + y(c(theta0[1],
theta0[2]-h,theta0[3],theta0[4],theta0[5]-h)))/(2*h^2) - ((sd.beta + sd.rho)/2)
        sd.sig2a.sig2b <- (y(c(theta0[1],
theta0[2],theta0[3]+h,theta0[4]+h,theta0[5]))-2*y(theta0) + y(c(theta0[1],
theta0[2],theta0[3]-h,theta0[4]-h,theta0[5]))))/(2*h^2) - ((sd.sig2a + sd.sig2b)/2)
        sd.sig2a.rho <- (y(c(theta0[1],
theta0[2],theta0[3]+h,theta0[4],theta0[5]+h))-2*y(theta0) + y(c(theta0[1],
theta0[2],theta0[3]-h,theta0[4],theta0[5]-h)))/(2*h^2) - ((sd.sig2a + sd.rho)/2)
        sd.sig2b.rho <- (y(c(theta0[1],
theta0[2],theta0[3],theta0[4]+h,theta0[5]+h))-2*y(theta0) + y(c(theta0[1],
theta0[2],theta0[3],theta0[4]-h,theta0[5]-h)))/(2*h^2) - ((sd.sig2b + sd.rho)/2)
        hess<-matrix(c(sd.alpha, sd.alpha.beta, sd.alpha.sig2a, sd.alpha.sig2b,
sd.alpha.rho, sd.alpha.beta, sd.beta, sd.beta.sig2a, sd.beta.sig2b, sd.beta.rho,
```

```
sd.alpha.sig2a, sd.beta.sig2a, sd.sig2a, sd.sig2a.sig2b, sd.sig2a.rho,
sd.alpha.sig2b, sd.beta.sig2b, sd.sig2a.sig2b, sd.sig2b, sd.sig2b.rho,
sd.alpha.rho, sd.beta.rho, sd.sig2a.rho, sd.sig2b.rho, sd.rho),5,5)
        hess.inv <- solve(hess)
            while (i<=N) {
                 fd.alpha<-(f(theta0[1]+h)-f(theta0[1]))/h
                 fd.beta<-(f1(theta0[2]+h)-f1(theta0[2]))/h
                 fd.sig2a<-(f2(theta0[3]+h)-f2(theta0[3]))/h
                 fd.sig2b<-(f3(theta0[4]+h)-f3(theta0[4]))/h
                   fd.rho<-(f4(theta0[5]+h)-f4(theta0[5]))/h
                 if(is.na(fd.alpha)==TRUE | is.na(fd.beta)==TRUE |
is.na(fd.sig2a)==TRUE | is.na(fd.sig2b)==TRUE | is.na(fd.rho)==TRUE | fd.alpha== -
Inf | fd.beta== -Inf | fd.sig2a== -Inf | fd.sig2b== -Inf | fd.rho== -Inf |
fd.alpha== Inf | fd.beta== Inf | fd.sig2a== Inf | fd.sig2b== Inf | fd.rho== Inf){
                     p[,i] <- theta.start
                     i<- i + 1
                     break
                   }
                   score <- c(fd.alpha, fd.beta, fd.sig2a, fd.sig2b, fd.rho)
                   theta1 <- theta0 - hess.inv%*% score
                   if(theta1[3] <= 0) {theta1[3] = theta.start[3]}
                   if(theta1[4] <= 0) {theta1[4] = theta.start[4]}
                   if(theta1[5] <= -1 | theta1[5] >= 1) {theta1[5] =
theta.start[5]}
                   if(abs(theta1[1]-theta0[1]) > 0.5) {theta1[1]=theta.start[1]}
                   if(abs(theta1[2]-theta0[2]) > 0.5) {theta1[2]=theta.start[2]}
                   if(abs(theta1[3]-theta0[3]) > 0.5) {theta1[3]=theta.start[3]}
                   if(abs(theta1[4]-theta0[4]) > 0.5) {theta1[4]=theta.start[4]}
                   if(abs(theta1[5]-theta0[5]) > 0.5) {theta1[5]=theta.start[5]}
                   p[,i] <- theta1
                   i <- i + 1
                   if (abs(theta1[1]-theta0[1]) < tol && abs(theta1[2]-theta0[2])
< tol && abs(theta1[3]-theta0[3]) < tol && abs(theta1[4]-theta0[4]) < tol &&
abs(theta1[5]-theta0[5]) < tol) break
                   if (any(duplicated(round(p[,1:(i-1)], digits = 6), MARGIN =
2))==TRUE) break
                   theta0 <- theta1
            }
            return(p[,1:(i-1)])
}
p <- OFI(mydata, y)
p

if(length(p)==5){OFI.theta=p}
if(length(p)>5){OFI.theta=p[,dim(p)[2]]}
OFI.theta
```
