# Supplementary material for: Maximum likelihood estimation based on Newton–Raphson iteration for the bivariate random effects model in test accuracy meta-analysis
Source: Stat Methods Med Res. 2019 Jun 11;29(4):1197–211. doi: 10.1177/0962280219853602 (PMC7221455; doi:10.1177/0962280219853602)
Supplement: Supplemental Material2 - Supplemental material for Maximum likelihood estimation based on Newton–Raphson iteration for the bivariate random effects model in test accuracy meta-analysis [file Supplemental_Material2.pdf]

Online Appendix 2 - Simulated data sets where *glmer* failed to converge

Example 1

k	TP	TN	FN	FP
**1**	22	96	5	13
**2**	28	119	4	9
**3**	27	159	15	11
**4**	21	159	20	5
**5**	19	90	6	11
**6**	37	164	5	7
**7**	18	66	2	16
**8**	4	18	1	2
**9**	10	33	1	13
**10**	9	66	8	4

Example 2

k	TP	TN	FN	FP
**1**	19	74	1	9
**2**	40	169	8	26
**3**	8	33	0	2
**4**	17	81	5	9
**5**	35	164	6	1
**6**	18	70	3	17
**7**	5	20	0	1
**8**	24	127	9	7
**9**	24	124	9	11
**10**	22	83	4	21

Example 3

k	TP	TN	FN	FP
**1**	36	178	11	11
**2**	33	139	8	25
**3**	22	68	1	24
**4**	3	39	6	0
**5**	26	150	14	11
**6**	14	64	4	10
**7**	7	32	1	1
**8**	14	67	3	3
**9**	22	108	8	15
**10**	21	143	15	3
